# Bioinspired Active Site with a Coordination-Adaptive Organosulfonate Ligand for Catalytic Water Oxidation at Neutral pH

**DOI:** 10.1021/jacs.3c03415

**Published:** 2023-05-17

**Authors:** Tianqi Liu, Shaoqi Zhan, Nannan Shen, Linqin Wang, Zoltán Szabó, Hao Yang, Mårten S. G. Ahlquist, Licheng Sun

**Affiliations:** † Department of Chemistry, School of Engineering Sciences in Chemistry Biotechnology and Health, KTH Royal Institute of Technology, 10044 Stockholm, Sweden; ‡ Department of Chemistry-BMC, Uppsala University, BMC Box 576, S-751 23 Uppsala, Sweden; § Department of Chemistry, University of Oxford, Oxford OX1 3QZ, U.K.; ∥ State Key Laboratory of Radiation Medicine and Protection, School for Radiological and Interdisciplinary Sciences (RAD-X) and Collaborative Innovation Center of Radiation Medicine of Jiangsu Higher Education Institutions, Soochow University, 215123 Suzhou, China; ⊥ Center of Artificial Photosynthesis for Solar Fuels and Department of Chemistry, School of Science, Westlake University, 310024 Hangzhou, China; # State Key Laboratory of Fine Chemicals, Dalian University of Technology (DUT), Dalian 116024, China

## Abstract

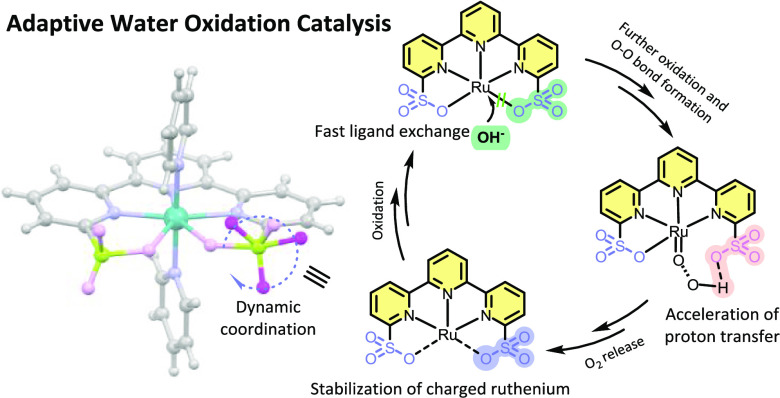

Many enzymes use adaptive frameworks to preorganize substrates, accommodate various structural and electronic demands of intermediates, and accelerate related catalysis. Inspired by biological systems, a Ru-based molecular water oxidation catalyst containing a configurationally labile ligand [2,2′:6′,2″-terpyridine]-6,6″-disulfonate was designed to mimic enzymatic framework, in which the sulfonate coordination is highly flexible and functions as both an electron donor to stabilize high-valent Ru and a proton acceptor to accelerate water dissociation, thus boosting the catalytic water oxidation performance thermodynamically and kinetically. The combination of single-crystal X-ray analysis, various temperature NMR, electrochemical techniques, and DFT calculations was utilized to investigate the fundamental role of the self-adaptive ligand, demonstrating that the on-demand configurational changes give rise to fast catalytic kinetics with a turnover frequency (TOF) over 2000 s^–1^, which is compared to oxygen-evolving complex (OEC) in natural photosynthesis.

## Introduction

**Figure 1:**
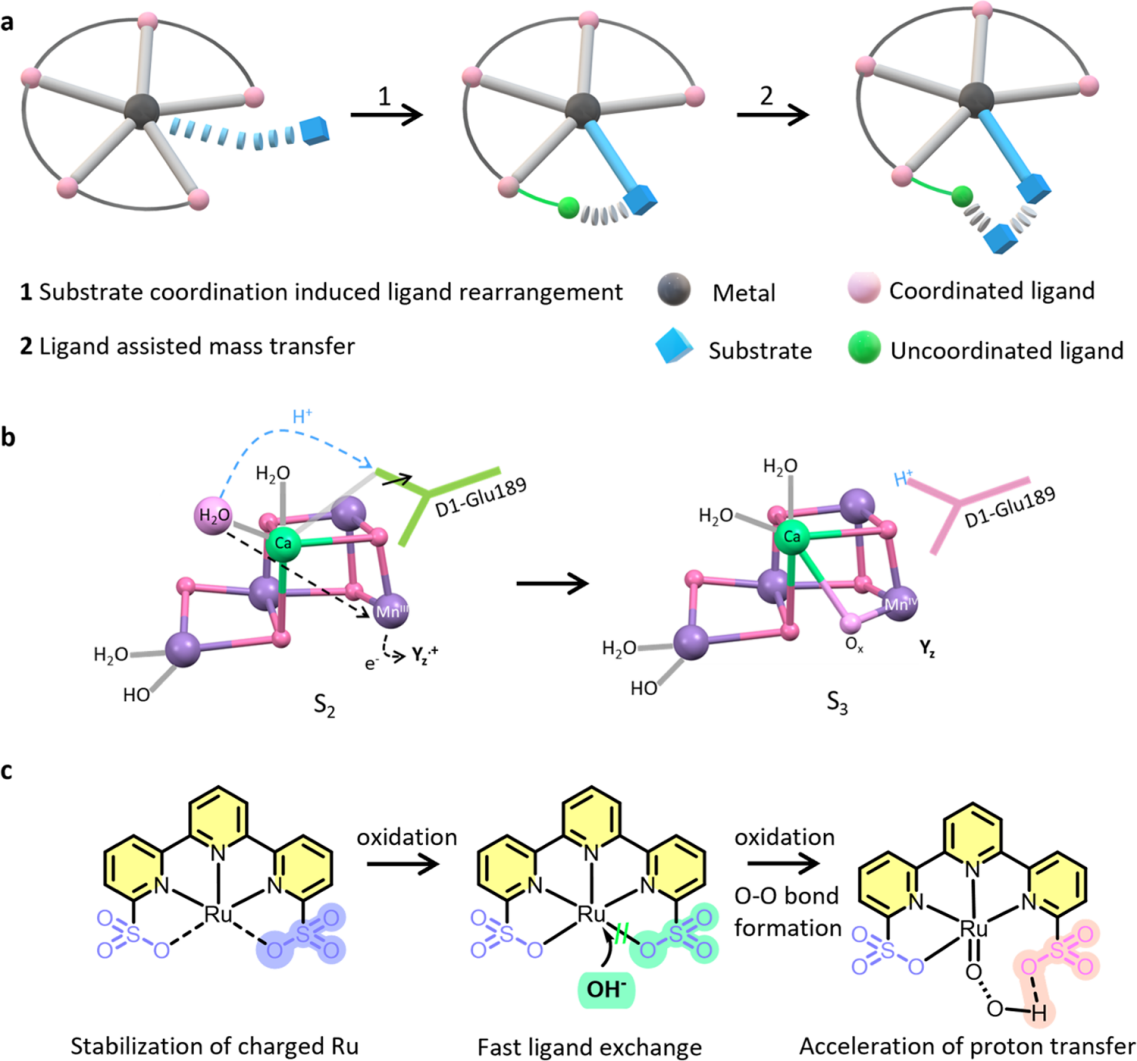
(a) Rearrangement of first and second coordination spheres during enzymatic catalysis. (b) Transition from S_2_ to S_3_ during the oxygen-evolving Kok cycle (O*_x_* stands for OH or O). (c) Proposed self-adaptive configurations during water oxidation catalysis by **Ru-tds**. Axial ligands are omitted for clarity.

The design of catalysts that rival the proficiency of metalloenzymes is an intense research topic of coordination chemistry.^1,2^ Enzymes catalyze reactions in a dynamic manner, binding substrates at the labile coordination site and releasing products by interconverting the conformations on different time scales (Figure 1a).^3−5^ Several crystallographic and time-resolved spectroscopic techniques have disclosed dynamic ligand exchanges in the vicinity of the catalytic site during photosynthesis,^6,7^ nitrogen fixation,^8^ oxygen reduction,^9,10^ DNA synthesis/cleavage,^11−13^ etc.^14^ For example, in the Kok cycle of photosystem II (PSII), the substrate water would rearrange from Ca to pentacoordinate Mn in the S_2_ → S_3_ transition accompanied by the oxidation of Mn from III to IV,^6,15−20^ during which the Ca-ligated D1-Glu189 residue moves away accordingly and makes space for the substrate water coordination (Figure 1b). The detached D1-Glu189 or/and other residues serve as proton acceptors, transferring protons from the catalytic center to bulk water.^6,21,22^

**Scheme 1:**
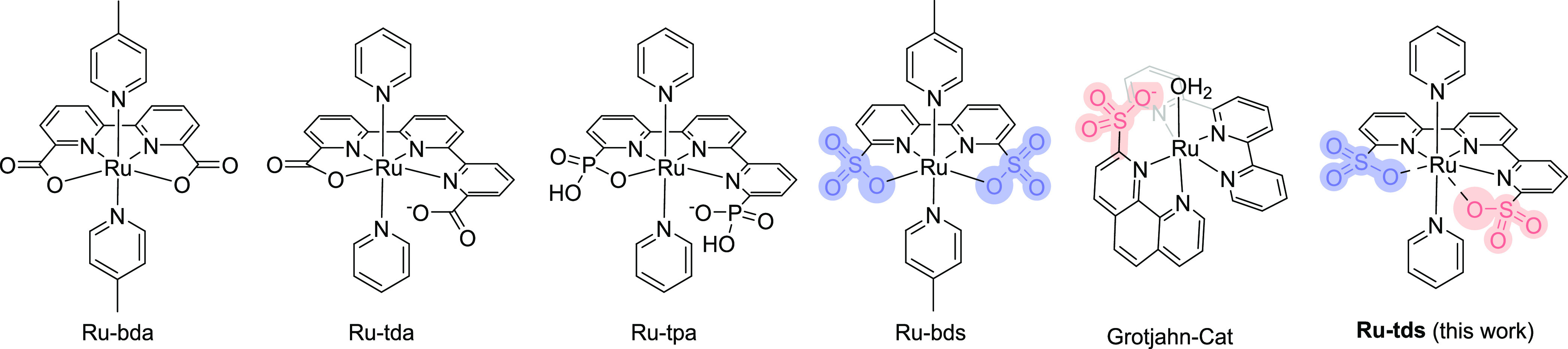
Structures of Water Oxidation Catalysts Discussed in the Paper and **Ru-tds**

To mimic the function of the adaptive architecture in PSII, conformationally flexible ligands with the ability to accommodate the structural and electronic demands of the different intermediates have been successfully applied in artificial water oxidation catalysts and have resulted in a few elegant examples of seven-coordination phenomena in **Ru-bda** and **Ru-tda** (Scheme 1, bda = 2,2′-bipyridine-6,6′-dicarboxylate, tda = [2,2′:6′,2″-terpyridine]-6,6″-dicarboxylate) catalysts that display enhanced activity comparable to the Mn_4_CaO*_x_* cluster of PSII.^23,24^ The classic **Ru-bda** system mediates O–O bond formation via the interaction of two metal-oxyl species (I2M), which is highly dependent on the interaction between the catalysts.^25^ Modifications on distal ligands to preorganize the substrate water can promote an alternative water nucleophilic attack (WNA) pathway, where the preorganized water network serves as a base to facilitate the proton transfer process.^26−28^ Another intriguing strategy is to introduce intramolecular proton acceptors by rearrangements of the coordination conformations, which is skilfully illustrated by **Ru-tda** and **Ru-tpa** type water oxidation catalysts (Scheme 1, tpa = 2,2′:6′,2″-terpyridine-6,6″-diphosphonate).^29−31^ However, installations of proton acceptor at the second coordination sphere inevitably lead to a competitive coordination with substrate water, making it impossible to fully leverage the catalytic site.^29,32^ Therefore, the coordination ability of the proton relay unit needs to be negotiated with the water molecule to lower the energy required for substrate binding and activation.^33,34^

Organosulfonates are a relatively unexplored type of ligand among the reported water oxidation catalysts^35−40^ due to their relatively weak coordination capabilities,^41−43^ while they offer potential applications in adaptive chemistry. As a proton acceptor, sulfonate is also able to accept up to six hydrogen bonds with the lone pairs of oxygen. Indeed, replacing the dicarboxylates of **Ru-bda** with disulfonates enables a 40-fold increase in water oxidation efficiency (Scheme 1, **Ru-bds**, bds = 2,2′-bipyridine-6,6′-disulfonate).^44^ Introduction of a remote sulfonate at the second coordination sphere also leads to a boosted performance in the context of higher-onset-potential Ru-tpy-type catalysts (around 800 mV, tpy = terpyridine), allowing for excellent performance under both acidic and basic conditions (Scheme 1, **Grotjahn-Cat**).^45,46^ Ideally, simultaneously introducing sulfonates to both the first and second coordination spheres can enrich the electron density of the metal, promote substrate binding, and accelerate the proton transfer process (Figure 1c), enabling fast water oxidation kinetics under a mild driving force.

In this work, a bioinspired catalyst with an adaptive architecture, **Ru-tds** (tds = [2,2′:6′,2″-terpyridine]-6,6″-disulfonate, Scheme 1), is designed to resemble enzymes, where the ligand can satisfy the varied electronic and geometric requirements of catalytic intermediates through dynamic sulfonate coordination/de-coordination. The catalyst achieves high TOFs over 2000 s^–1^ with a mild onset potential of 530 mV and an overpotential of 620 mV under neutral conditions, which is compared to the Mn_4_CaO*_x_* cluster of PSII. Spectroscopic and kinetic studies in concert with computational results reveal that the proton transfer events in the catalytic cycle are fast enough; as such, the rate-determining step (RDS) shifts to the substrate binding process via aqua-sulfonate ligand exchange.

## Experimental Section

### Synthesis and Characterization

The ligand [2,2′:6′,2″-terpyridine]-6,6″-disulfonic acid (**H**_**2**_**tds**) was synthesized in two steps as described in the Supporting Information. In short, 6,6″-dibromo-2,2′:6′,2″-terpyridine was initially transformed into [2,2′:6′,2″-terpyridine]-6,6″-dithiol via a nucleophilic aromatic substitution reaction, followed by oxidation of the dithiol to disulfonic acid. Complex **Ru-tds** was prepared via a one-pot reaction, *i.e.*, refluxing **H**_**2**_**tds**, [Ru(DMSO)_4_Cl_2_], and pyridine in ethanol under N_2_. The desired catalyst was isolated via column chromatography and characterized by nuclear magnetic resonance (NMR) and high-resolution mass spectrometry (HRMS) (Figures S1–S6). Two byproducts were also isolated by column chromatography and characterized by ^1^H NMR, which are tentatively assigned to Ru(tds)_2_ and Ru(tds)(py)(DMSO), respectively (Figure S22).

## Results and Discussion

**Figure 2:**
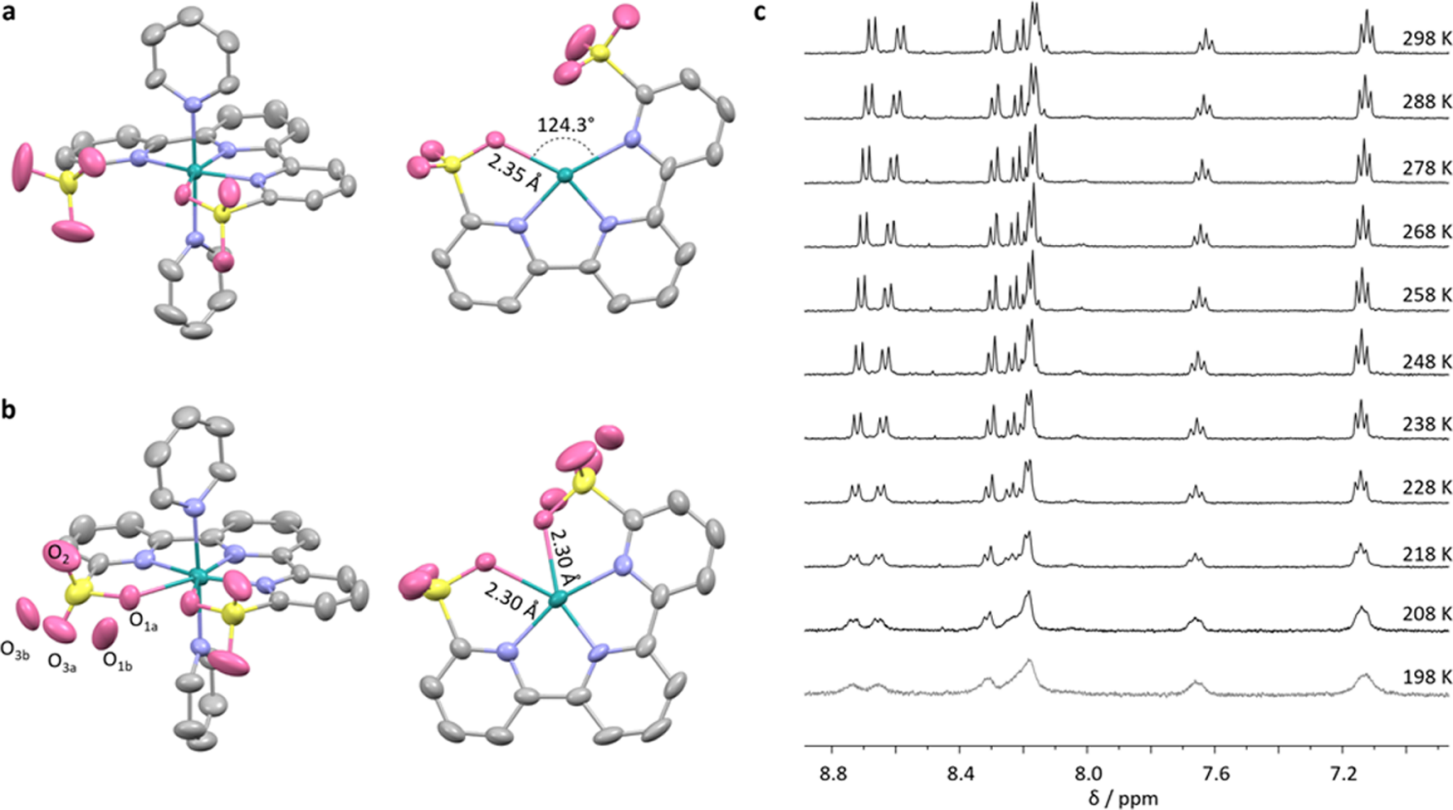
Single-crystal structures of (a) **Ru**^**II**^**(tds-κ-N**^**3**^**O)Py**_**2**_ and (b) **Ru**^**II**^**(tds-κ-N**^**3**^**O**^**2**^**)Py**_**2**_ with thermal ellipsoids at 50% probability. Hydrogen atoms and solvent molecules are omitted for clarity. (c) VT ^1^H NMR spectra of **Ru-tds** in CD_3_OD/D_2_O (v/v = 4/1).

Two conformations of the single-crystal structure, *i.e.*, **Ru**^**II**^**(tds-κ-N**^**3**^**O)Py**_**2**_ and **Ru**^**II**^**(tds-κ-N**^**3**^**O**^**2**^**)Py**_**2**_, were obtained in different batches of crystal growth (Figure 2, CCDC: 2209276 and 2209277). Complex **Ru**^**II**^**(tds-κ-N**^**3**^**O)Py**_**2**_ features a distorted octahedral geometry with a dangling sulfonate at the second coordination sphere. The N_pyridine_–Ru–O_sulfonate_ angle is 124.3°, which is slightly larger than that of carboxyl-containing analogues **Ru-tda** and **Ru-bda**(29,47) and much larger than that of phosphonate-containing (**Ru-tpa**) and sulfonate-containing (**Ru-bds**) analogues (Figure 2a and Table 1).^30,44^ The large angle can serve as the site for water binding, activation, and O–O bond formation. Complex **Ru**^**II**^**(tds-κ-N**^**3**^**O**^**2**^**)Py**_**2**_ exhibits a pentagonal bipyramidal coordination geometry with splitting positions of oxygen atoms (O_1a/1b_ and O_3a/3b_, Figure 2b), suggesting an alternate position of the sulfonate. To the best of our knowledge, this is the first isolated pseudo-seven-coordinated Ru(II) complex. It should be noted that the seven-coordinate Ru(II) complex (20-electron rule) is thermodynamically unstable due to the violation of the 18-electron rule. Collectively considering the long Ru–O distance (2.30–2.35 Å) and the splitting positions, it is hypothesized that sulfonates in both **Ru**^**II**^**(tds-κ-N**^**3**^**O)Py**_**2**_ and **Ru**^**II**^**(tds-κ-N**^**3**^**O**^**2**^**)Py**_**2**_ are weakly bound. The weak sulfonate coordination can promote substrate binding in the subsequent catalysis step via ligand exchange.

**Table 1:**
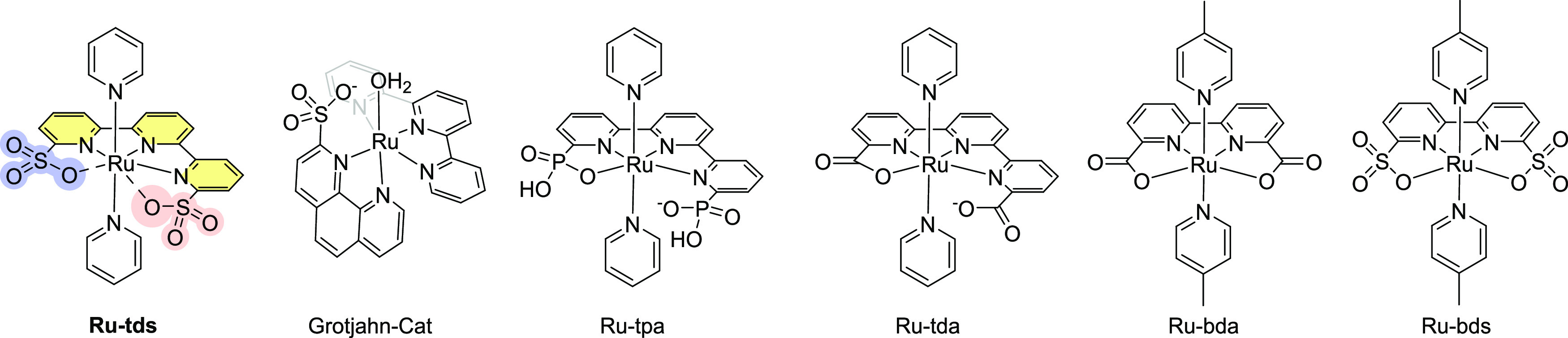
Crystal and Catalysis Data for **Ru-tds** and Other State-of-the-art Reference Catalysts

		**Ru-tds**	Grotjahn-Cat^45^	**Ru-tpa**(30)	**Ru-tda**(29)	**Ru-bda**(47)	**Ru-bds**(44)
Ru–O (Å)^a^		2.35	2.18	2.19	2.14–2.20	2.17–2.21	2.19–2.21
O–Ru–O(N) (°)^a^		124.3		114.6	122.4	123.0	114.7–115.1
onset potential (V)^b^		1.76	2.02	1.76	1.70–1.80	1.55	1.65
overpotential (V)^b^		0.62	0.80	0.69	0.64	0.21/0.67	0.62
TOF (s^–1^)	method A^c^	2239 ± 311					
	method B^d^	3242	2598				
	method C^e^	4195				300	12 900
TOF_max_ (s^–1^)^f^		12 000		16 000	8000		
mechanism^g^		WNA	WNA	WNA	WNA	I2M	I2M

a Data for bond length and angle extracted from their crystal structures.

b Values of onset potential vs RHE extracted from their CVs at pH 7; values of overpotential (*E*_cat/2_) extracted from their CVs at pH 7 according to the suggested method in the literature,^49^ and the two values for Ru-bda are extracted from two “catalytic plateau,” respectively.^44,50^

c TOF stands for turnover frequency, values extracted from their CVs at pH 7 according to eq 3 in the Supporting Information.

d Values estimated according to eq 2 in the Supporting Information and the reference (45) at a scan rate of 0.01 V s^–1^ at pH 7.

e Values estimated according to eq 4 in the Supporting Information and the reference (44) at a scan rate of 0.1 V s^–1^ at pH 7.

f TOF_max_ stands for turnover frequency maximum that was estimated by the foot of the wave analysis^29^ at pH 7 (for details, see Figure S14). For **Ru-tpa** and **Ru-tda**, the values were calculated after catalyst activation.

g WNA stands for water nucleophilic attack, and I2M stands for interaction of two metal-oxo.

The ^1^H NMR spectra show that **Ru-tds** maintained its symmetry in solution, in contrast to the asymmetrical conformation found in the single crystal. The ^1^H NMR spectrum shows only three signals for the axial ligands and five signals for the equatorial ligand at room temperature (298 K, Figure 2c), which suggests a fast dynamic coordination behavior of the sulfonate groups. The dynamic coordination was then investigated by recording ^1^H NMR spectra at lower temperatures in a mixed solvent (CD_3_OD/D_2_O, v/v = 4/1) until its freezing point was reached (ca. 198 K). In contrast to the observations for the **Ru-tpa** analogue containing two phosphate groups,^30^ the spectra did not change with temperature (Figure 2c). Even below 200 K, one set of signals can be observed for the ligands, indicating a fast chemical exchange on the NMR chemical shift time scale, that is, a fast coordination/de-coordination of the sulfonate groups. This rate is much faster than that observed for phosphate coordination/de-coordination in the **Ru-tpa** system, where separate signals of the ligand can be observed below 253 K. In our view, the line broadening of the signals observed below 218 K is due to the fast transverse relaxation (T2) caused by the increased viscosity of the solvent.

### Electrochemical Studies

**Figure 3:**
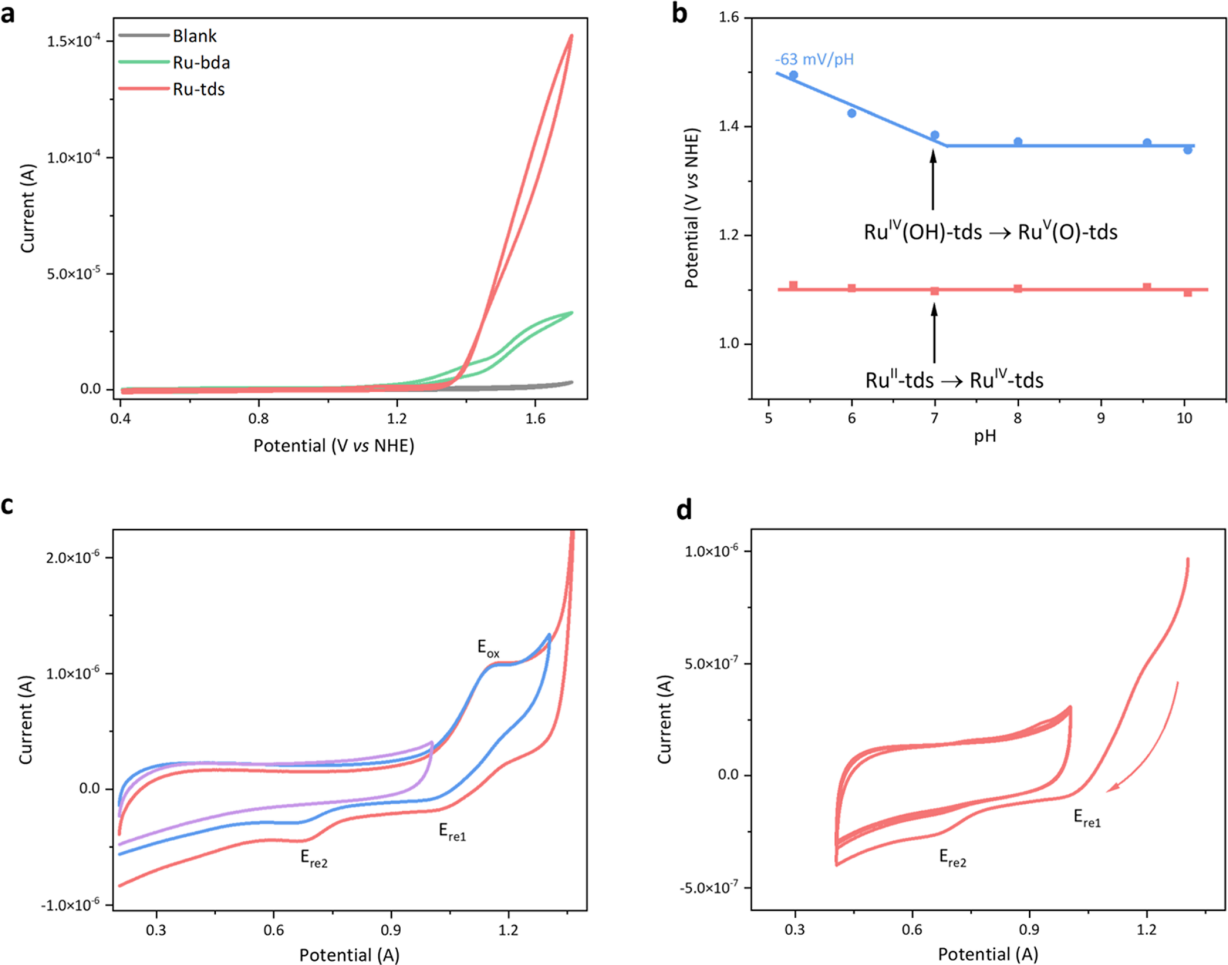
(a) CVs without background subtraction of 0.13 mM **Ru-tds** and **Ru-bda** at pH 7.0 in a 0.1 M phosphate buffer solution containing 1% CF_3_CH_2_OH, scan rate = 100 mV s^–1^, working electrode: BDD. (b) Potential vs pH diagram for **Ru-tds** in aqueous buffer solutions containing 1% CF_3_CH_2_OH, in which the potentials of Ru^V/IV^ were measured at a current of 3.7 μA from their LSVs. (c) CVs without background subtraction of **Ru-tds** at different potential windows, working electrode: BDD. (d) Negative-scan CV without background subtraction from 1.3 V, working electrode: BDD.

The electrochemical properties of **Ru-tds** were investigated by cyclic voltammetry (CV) at pH 7.0 in a 0.1 M phosphate buffer solution containing 1% CF_3_CH_2_OH. A pH-independent oxidation peak (Figure 3c) was observed at *E*_ox_ = 1.1 V, which can be assigned to a pure electron transfer process and will be discussed later. The CVs at different scan rates indicate that *E*_ox_ is a diffusion-controlled electrochemical process, demonstrating a linear relationship between peak currents and the square root of scan rates (Figures S10 and S11). Subsequently, a substantial enhancement of the catalytic peak at 1.35 V vs NHE (onset potential) was observed, followed by a large catalytic current density of 4.85 mA cm^–2^ at 1.7 V by using a boron-doped diamond electrode (BDD, 0.0314 cm^2^, Figure 3a) as the working electrode. Under the same experimental conditions, the current density of the reference catalyst **Ru-bda** is 4.5 times lower (1.06 mA cm^–2^, Figure 3a) than that of **Ru-tds**.

The differential pulse voltammograms (DPVs) of **Ru-tds** at various pH values were measured to study the proton and electron transfer processes during water activation and O–O bond formation (Figures 3b and S7). A pH-independent oxidation process at 1.10 V appears in the pH range of 5–10. Since Ru species generated during this process cannot trigger water oxidation, we further extracted the potentials of higher valent species from their catalytic peaks (details for the potential determination can be found in the Note below Figure S7). The catalytic currents are pH-dependent with a slope of −0.063 V/pH (Figure 3b, blue) in the pH range of 5–7, suggesting that a 1H^+^/1e^–^ transfer process occurs before O–O bond formation, most likely on Ru^V/IV^. Accordingly, the first procedure at 1.1 V should be a 2e^–^ removal process as the catalyst precursor is in the Ru^II^ state. Our conclusion is in agreement with the fact that the analogue catalyst **Ru-tda** precursor (without an aqua ligand) can be oxidized to the Ru^IV^ state around 1.1 V in the same pH window.^29^

**Scheme 2:**
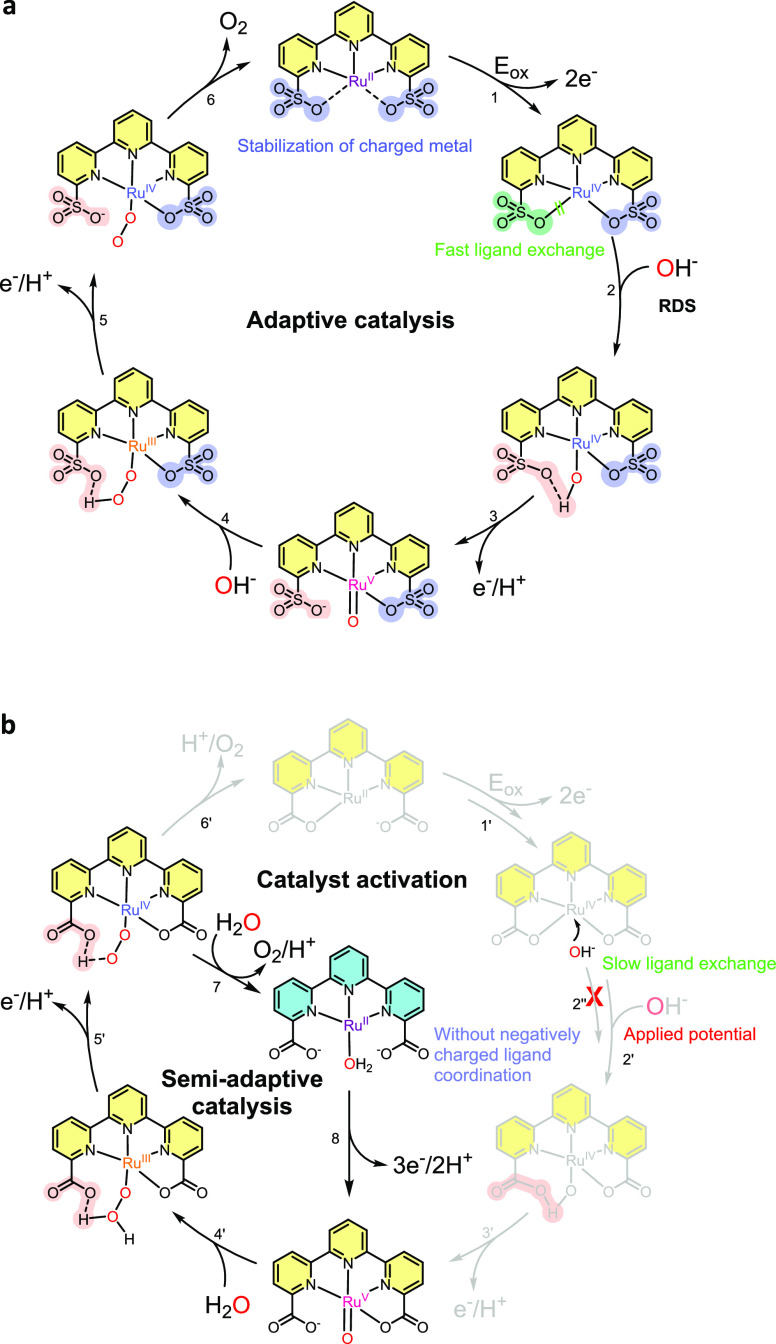
Proposed Water Oxidation Pathways of (a) Adaptive Catalysis by **Ru-tds** and (b) Semiadaptive Catalysis by the Analogue Catalyst^,^^29^ Axial ligands are omitted for clarity.

The water activation mechanism by **Ru-tds** is proposed in Scheme 2a based on the electrochemical data mentioned above. **Ru**^**II**^**-tds** is initially oxidized to **Ru**^**IV**^**-tds**, followed by the formation of seven-coordinated **Ru**^**IV**^**(OH)-tds** as suggested by DFT calculation that will be discussed later (Figures 5, S20, and S21). Sulfonate is generally regarded as a poor ligand in terms of coordination ability, as indicated by its role in most metal aqua complexes being noncoordinating counter ions.^41^ Consequently, the Ru-aqua complex can be readily formed via ligand exchange. **Ru**^**IV**^**(OH)-tds** then undergoes a 1H^+^/1e^–^ transfer process to generate **Ru**^**V**^**(O)-tds** species.

Two reduction peaks appear at *E*_re1_ = 1.02 V and *E*_re2_ = 0.67 V, respectively, in the reverse CV scan (Figure 3c), which are assigned to consecutive one-electron reduction of the Ru-aqua species generated at higher potentials. This assumption is supported by the following evidence. First, reduction peaks only manifest once the Ru^IV^ state is reached (Figure 3c, purple), demonstrating that the reduction waves *E*_re1_ and *E*_re2_ are connected to the oxidation wave *E*_ox_. Second, the peak currents at *E*_re1_ and *E*_re2_ decrease as the scan window narrows (Figure 3c, red and blue), while the peak current at *E*_ox_ remains the same, suggesting that the Ru-aqua complex is preferred at higher potentials and longer time scales. Third, the backward scan in Figure 3d shows that the two reduction peaks gradually vanish in the subsequent CVs in a smaller window, indicating that these two signals originate from diffusible active species rather than species adsorbed on the electrode surface due to catalyst decomposition. Fourth, the in situ formation of new active Ru-aqua species is indicated by the curve crossing between the forward and backward scans^29,48^ under a high scan rate (100 mV s^–1^, Figure 3a). This crossover disappears as the scan rate decreases (10 mV s^–1^, Figure S15) because the longer time scale results in the maximum conversion of **Ru-tds** to the corresponding catalytically active Ru-aqua species in the electrical double layer. Fifth, the potentials of *E*_re2_ are pH-dependent with a slope of −0.051 V/pH (Figure S16) in the pH range of 5–8, validating the formation of Ru-aqua species. Finally, the analogue catalyst **Ru(H**_**2**_**O)-tda** also showed reduction potentials similar to those of Ru^III/IV^ and Ru^II/III^ (0.93 and 0.72 V) at pH 7.^29^

### Catalytic Performance

The generation of oxygen was confirmed by controlled potential electrolysis (1.7 V vs NHE) at pH 7 and monitored by a pressure transducer. The average Faradaic efficiency for water oxidation is over 92%, indicating that the majority of gas produced is oxygen (Figure S19). The linear relationship between catalytic current and catalyst concentration (Figures S8 and S9) and scan rate-independent catalytic current (Figures S12 and S13) enables us to evaluate the catalytic TOF by eq 3 in the Supporting Information, which provides a reliable method to compare the TOF of **Ru-tds** with the majority of reported catalysts.^51−54^ The TOF value of 2239 ± 311 s^–1^ is compared to that of OEC in PSII and among the highest activities reported thus far for Ru- and non-noble metal-based catalysts.^55^ In addition, other modified methods are also used to fairly compare activities with reported state-of-the-art catalysts. (1) The sulfonate at the first coordination sphere is designed to enrich the electron density of Ru and to obtain a lower onset potential and overpotential. Indeed, the lower onset potential/overpotential and higher TOF are attained for **Ru-tds** compared to **Grotjahn-Cat** (Tables 1 and S2), in which the sulfonate is located only at the second coordination sphere.^45^ (2) We also estimated the TOF_max_ value of 12 000 s^–1^ according to the foot of the wave analysis (FOWA, Figure S14), however, this method assumes a scenario in which no side phenomena are operative. Instead, **Ru-tds** attained a comparable current density at a lower concentration than **Ru-tda** (0.13 vs 0.45 mM^29^), demonstrating that sulfonate at the second coordination sphere has a superior capacity for proton transfer than carboxylate. More importantly, the aqua-carboxylate exchange kinetics are slow (step 2″, Scheme 2b); as a consequence, an electrolysis-based activation procedure to generate the catalytically active species is necessary for **Ru-tda** (step 2′, Scheme 2b).^29^ This procedure is not required for **Ru-tds** due to the flexible coordination ability of sulfonates (step 2, Scheme 2a). The negatively charged sulfonates can re-coordinate to stabilize the charged metal center after oxygen release for **Ru-tds** (step 6, Scheme 2a) to close the adaptive catalytic cycle, whereas the semiadaptive **Ru-tda** catalyst either generated new active species with only neutral ligands (step 7, Scheme 2b) or returned to the catalyst precursor (step 6′, Scheme 2b). After long-term electrolysis, a new oxidation signal at 0.9 V with a relatively weak peak current appeared (inset, Figure 6a), suggesting a possible alternative pathway to generate **Ru**^**II**^**(OH**_**2**_**)-tds** (Scheme S1). (3) The **Ru-tpa** also shared a semiadaptive catalytic process and underwent more complicated structural transformations to generate the catalytically active species **Ru-tpaO** and **Ru-bpc** and other catalytically inactive species under high potentials (Table S2).^30,31,56^ (4) Although **Ru-bds** exhibits higher catalytic activity than **Ru-tds**, this is because **Ru-bds** catalyzes water oxidation via the I2M mechanism, where the TOF is proportional to the catalyst concentration (TOF_I2M_ = *k* [cat], TOF_WNA_ = *k*).^23^ Catalyst with the WNA mechanism is more promising for practical applications, such as immobilization on the electrode surface.

### Cation, Anion, and Kinetic Isotope Effects

**Figure 4:**
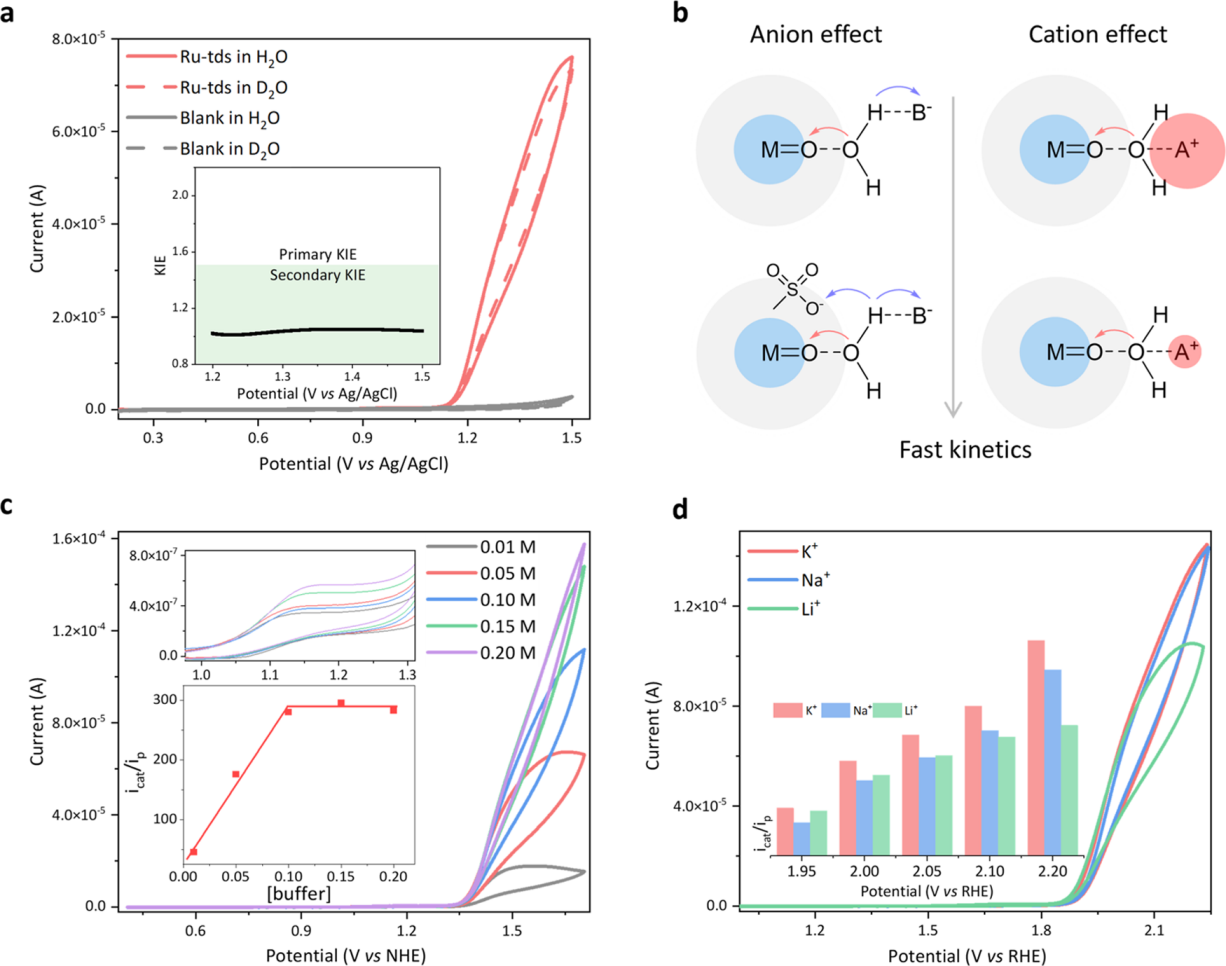
(a) CVs without background subtraction of 0.13 mM **Ru-tds** in a 0.1 M phosphate buffer solution in H_2_O and D_2_O, (pH = 7 and pD = 7.87) containing 1% CF_3_CH_2_OH, scan rate = 20 mV s^–1^, working electrode: BDD. (b) Schematic diagram of potential anion and cation effects for water oxidation. (c) CVs without background subtraction of 0.13 mM **Ru-tds** in phosphate buffer solution with various concentrations (pH 7.1) containing 1% CF_3_CH_2_OH, scan rate = 20 mV s^–1^, working electrode: BDD; inset: enlargement of the 1.0–1.3 V range (upper) and a plot of *i*_cat_/*i*_p_ vs [phosphate] (bottom). Ionic strength kept at 0.5 M with NaClO_4_. (d) CVs without background subtraction of 0.13 mM **Ru-tds** in 0.1 M KPi, NaPi, and LiPi, working electrode: BDD; inset: comparison of *i*_cat_/*i*_p_ at different potentials, scan rate = 20 mV s^–1^.

More insights into the catalytic mechanism of **Ru-tds** were obtained by cation, anion, and kinetic isotope effect (KIE) analysis. The catalytic current for water oxidation is linearly dependent on the concentration of the catalyst (Figures S8 and S9), suggesting that the RDS should take place on a single site in accordance with a WNA pathway. Since the O–H bond cleavage was implicated in steps 3 and 5, as proposed in Scheme 2a, the possibility that these steps were the RDS can be excluded according to a secondary KIE generated from the CV scans (Figure 4a). The negligible pH-dependent catalytic currents (*i*_cat_/*i*_p_) further support that the proton transfer is not involved in the RDS (Figure S17). Additionally, due to the varying solvation strengths (Li^+^ > Na^+^ > K^+^, Figure 4b), the nucleophilic attack ability of water is correlated with the types of cations utilized in electrolytes.^57,58^ The CVs were then measured in 0.1 M LiPi, NaPi, and KPi buffer solutions, as shown in Figure 4d, with insignificant cation effects in the potential range of 1.95–2.05 V against RHE, indicating that step 4 is not involved in RDS. The oxygen release (step 6) is significantly less demanding than the O–O bond formation from a free energy point of view.^59^ Collectively, the RDS should be the formation of Ru-aqua species (step 2, Scheme 2a). It is interesting to note that we also discovered relatively high KIE values in a backward scan LSV (Figure S18) and relatively obvious cation effects at the potentials of 2.10 and 2.20 V (Figure 4d), suggesting that the ligand exchange to produce Ru-aqua species is accelerated under the higher potential and longer time scale, resulting in the RDS being transferred somehow from step 2 to the step where the O–O bond formation takes place. Besides, as the buffer concentration increased from 0.01 to 0.10 M, base-enhanced water oxidation is observed (Figure 4c). The catalytic current reached a plateau as the buffer concentration increased further, whereas the anion can affect the catalytic activities of **Ru-bda** and **Ru-bds** over a broader range (0.01–0.20 M) due to the different RDS involved (proton-coupled oxidation step under the same conditions).^44,50^ Therefore, the involvement of the buffer in step 2 contributes to the faster aqua-sulfonate ligand exchange. In summary, the RDS can be accelerated via the maximum formation of Ru-aqua species in the presence of concentrated buffer solution under higher potentials and longer time scales.

### DFT Study on RDS

**Figure 5:**
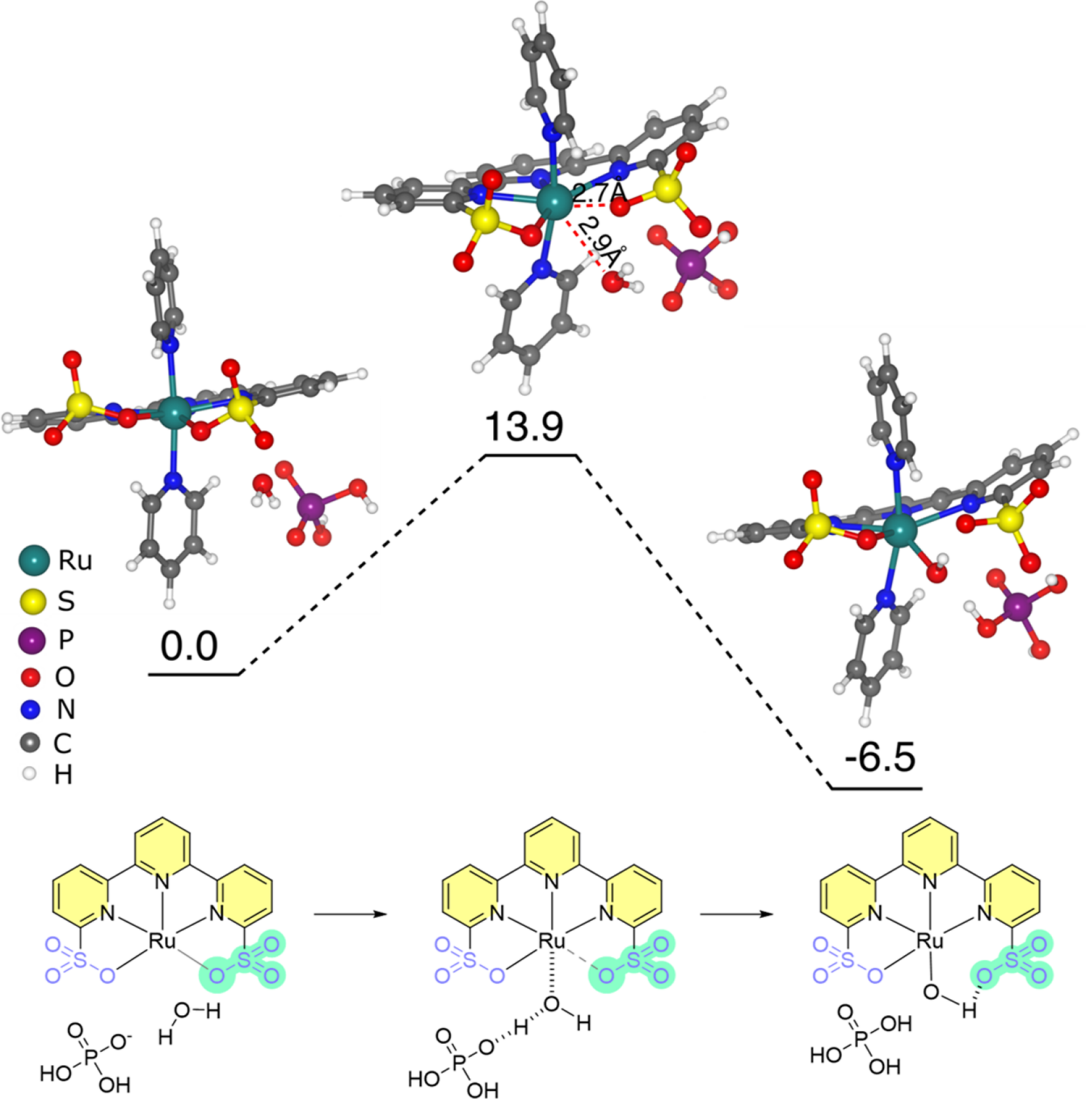
Energy profiles of ligand exchange on Ru^IV^ at pH 7.0 using H_2_PO_4_^–^ as the base. The units of energies are kcal mol^–1^.

DFT calculations were performed to elucidate the high performance of the electrochemical-driven water oxidation by **Ru-tds** at pH 7.0 (Figure 5). The calculated potential from the optimized 6-coordinate Ru^II^ species to 7-coordinate Ru^IV^ (Figure S20) is 1.25 eV, which is in good agreement with the experimental redox potential. In the 7-coordinate Ru^IV^, the O–Ru–O angle is only 68.6°, which could increase the barrier of the aqua ligand coordination to the Ru atom. This step is also tested as the RDS from the experiment; therefore, the detailed calculations were focused on this step. At an aqueous solution, the water coordination is endergonic with an activation free energy of 19.3 kcal mol^–1^ (Figure S21). While in the phosphate buffer, H_2_PO4^–^ (dominant species in pH 7.0 phosphate buffer) could stabilize the transition state by forming the H-bond and further taking the proton from a water molecule, leading to a much lower activation free energy of 13.9 kcal mol^–1^. The reaction is exergonic and generates a structure, where the OH forms the H-bond with the sulfonate group. This low activation free energy is consistent with a high TOF value. Hence, the buffer-promoted performance of **Ru-tds** can be ascribed to the fact that the buffer molecules facilitate the deprotonation of water to form OH^–^, accelerating the kinetics of the ligand exchange.

### Catalyst Stability

**Figure 6:**
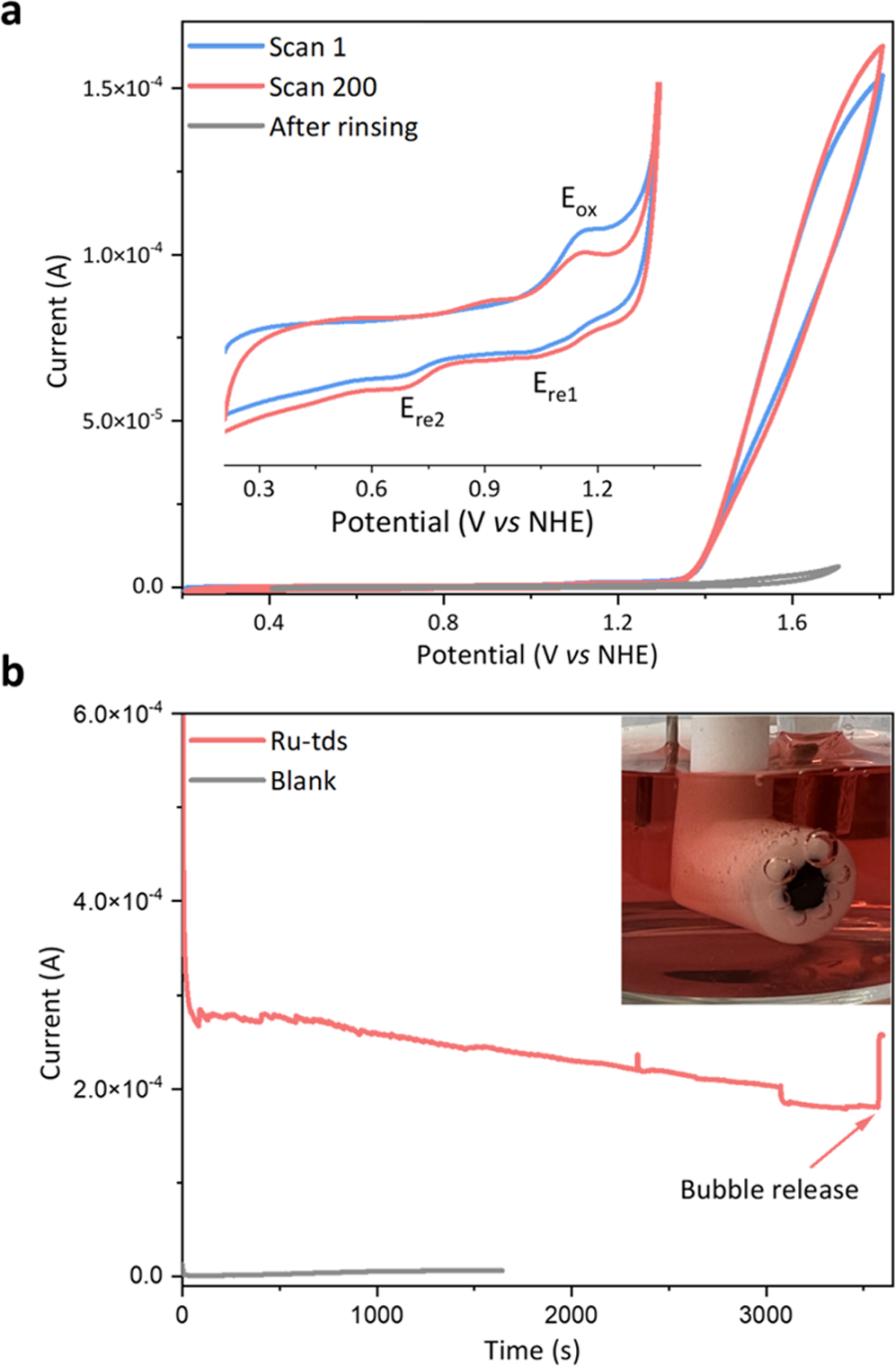
(a) 1st and 200th CV scans without background subtraction of **Ru-tds** at pH 7.0 in a 0.1 M phosphate buffer solution containing 1% CF_3_CH_2_OH, scan rate = 100 mV s^–1^, [**Ru-tds**] = 0.13 mM, working electrode: BDD; inset: enlargement of the 0.2–1.4 V range. (b) Controlled potential electrolysis (1.7 V) in a 0.1 M phosphate buffer solution containing 1% CF_3_CH_2_OH with **Ru-tds** (0.13 mM, red) and only with the electrode (gray), working electrode: GC; inset: bubble formation on the electrode surface.

The stability of **Ru-tds** for water oxidation is monitored via repetitive CV and controlled potential electrolysis (CPE) under neutral conditions (Figure 6). To test steady-state durability, the catalyst was first subjected to 5-cycle CV scans to generate enough Ru-aqua species. Figure 6a shows that the catalytic current increases slightly after 200 cycles of CV scan, which is due to the increased amount of Ru-aqua active species in the electrical double layer, as indicated by the decreased intensity of *E*_ox_ and increased intensity of *E*_re1_ and *E*_re2_. Following numerous CV scans, the electrode was removed from the solution, rinsed with water, and inserted into a brand-new electrolyte solution devoid of catalyst. The disappearance of redox and catalytic signals (gray, Figure 6a) indicates that no active species were deposited on the electrode surface throughout the whole stability tests. The CPE at 1.7 V vs NHE demonstrates that the current decreases gradually over time, which is a result of oxygen bubble formation on the electrode surface (Figure 6b). The catalytic current can be recovered by gently tickling the electrode to dislodge the bubbles. Taken together, these findings strongly suggest that **Ru-tds** functions as a reliable and efficient homogeneous molecular water oxidation catalyst at neutral pH.

## Conclusions

To conclude, the development of water oxidation catalysts that can mimic the dynamic catalytic nature of enzymes presents a timely challenge. The introduction of sulfonates to the ruthenium complex created labile coordination spheres: (1) the coordination of negatively charged sulfonates enriches the electron density of ruthenium, thus thermodynamically stabilizing the positively charged catalytic site at the initial state; (2) the dynamic sulfonate coordination/de-coordination creates an open site for water binding and enables the immediate formation of the Ru-aqua active species via aqua-sulfonate ligand exchange without an extra driving force, which is indispensable for the subsequent O–O bond formation; (3) substrate water absorption also results in a dangling and non-coordinated sulfonate for the kinetic acceleration of the proton transfer process; and (4) the dangling sulfonate re-coordinates to the ruthenium after the product oxygen releases to further stabilize the charged catalytic site. Consequently, high TOFs (2000–4000 s^–1^) were obtained with a mild onset potential of 530 mV and an overpotential of 620 mV. The dynamic nature of the **Ru-tds** catalyst has been proven by the combination of single-crystal X-ray analysis, VT NMR, electrochemical techniques, and theoretical studies. The introduction of labile sulfonate may provide a general strategy for homogeneous and even heterogeneous water oxidation catalysis and other related proton-coupled electron transfer reactions.

## Data Availability

Crystallographic data for the structures reported in this Article have been deposited at the Cambridge Crystallographic Data Centre under deposition numbers CCDC 2209276 and 2209277. Copies of the data can be obtained free of charge from www.ccdc.cam.ac.uk/structures/. Other data that support the findings of this study are available from the corresponding author upon reasonable request.

## Supporting Information Available

Experimental procedures; compounds synthesis and characterization (NMR, HRMS, and crystal data); electrochemical data; and DFT calculations (PDF)
