# Supplementary material for: Bioinspired Active Site with a Coordination-Adaptive Organosulfonate Ligand for Catalytic Water Oxidation at Neutral pH
Source: J Am Chem Soc. 2023 May 17;145(21):11818–28. doi: 10.1021/jacs.3c03415 (PMC10236490; doi:10.1021/jacs.3c03415)
Supplement: Supplementary file 1 — ja3c03415_si_001.pdf [file ja3c03415_si_001.pdf]

Supporting Information for

Bio-inspired Active Site with a Coordination-adaptive Organosulfonate Ligand for Catalytic Water Oxidation at Neutral pH

Tianqi Liu[1#], Shaoqi Zhan[2,3#], Nannan Shen[4#], Linqin Wang[5], Zoltán Szabó[1], Hao Yang[1], Mårten S. G. Ahlquist[1] and Licheng Sun[1,5,6*]

[1] Department of Chemistry, School of Engineering Sciences in Chemistry Biotechnology and Health, KTH Royal Institute of Technology, 10044 Stockholm, Sweden

[2] Department of Chemistry-BMC, Uppsala University, BMC Box 576, S-751 23 Uppsala, Sweden

[3] Department of Chemistry, University of Oxford, Oxford, OX1 3QZ, UK.

[4] State Key Laboratory of Radiation Medicine and Protection, School for Radiological and Interdisciplinary Sciences (RAD-X) and Collaborative Innovation Center of Radiation Medicine of Jiangsu Higher Education Institutions, Soochow University, 215123 Suzhou, China

[5] Center of Artificial Photosynthesis for Solar Fuels and Department of Chemistry, School of Science, Westlake University, 310024 Hangzhou, China

[6] State Key Laboratory of Fine Chemicals, Dalian University of Technology (DUT), Dalian 116024, China.

* Corresponding author: lichengs@kth.se

[#] These authors contributed equally: Tianqi Liu, Shaoqi Zhan and Nannan Shen

General Procedures

All chemicals were purchased from commercial suppliers (Jilin Yanshen Technology Co. Ltd., Merck, TCI, and Fisher Scientific Sweden) and used as received. All the solvents were purchased from Fisher Scientific Sweden and used as received. High resolution mass spectrometry was performed at the Westlake University. ^{1}H NMR and ^{13}C NMR spectra of the compounds were recorded with either a Bruker Ascend 400 or a Bruker Avance DMX 500 NMR spectrometer. Electrochemistry measurements were carried out with a CHI650 potentiostat, with either boron-doped diamond (BDD, Φ = 1 mm) electrode or glassy carbon (GC, Φ = 3 mm, used for controlled potential electrolysis only as shown in Figure 6b) electrode as working electrode, Pt wire as auxiliary electrode and measured versus Ag/AgCl/Sat. KCl as reference electrode. The porous glassy carbon electrode (1 x 1 cm) as the working electrode, Pt mesh as auxiliary electrode were used for Faradaic efficiency measurement. Details for electrode polishing: A small amount of alumina powder was placed on the polishing pad and diluted with a minimal amount of deionized water. The electrode was then polished by hand using a figure eight motion for at least 30 seconds. Once compete, rotate the disk electrode 1/4 of a turn, and repeat the figure eight motion, in the opposite direction for an equal amount of time, to ensure an even polish. Once polishing is complete, the electrode was thoroughly rinsed with deionized water to remove all the alumina.

Onset potential and overpotential determination

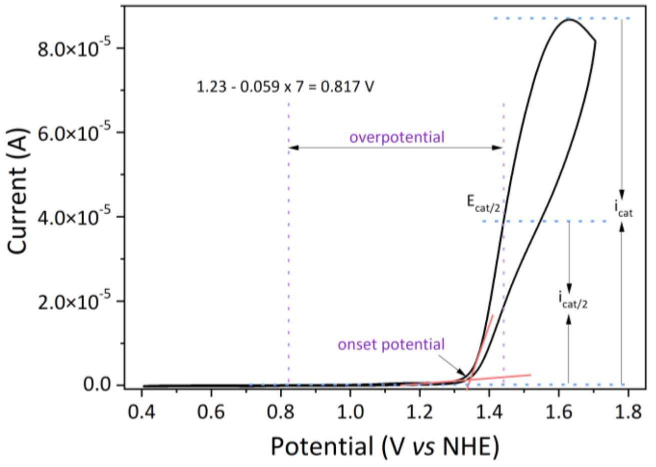

The onset potentials and overpotentials are determined according to Appel and Helm method.[1] The picture above shows an example of potential determinations at pH 7.

TOF values calculation from electrochemical experiments

Generally, the scan rate independent TOF can be obtained by taking the slope of i_{cat}/i_p *vs* 1/sqrt(v) (Figure S13 as an example) and applying it to equation 3, then solving for k_{cat}. Please see details below.

Method A: Randles–Ševčík equation describes the effect of scan rate on the peak current (i_p) for reversible electrochemical couples.

$$i_p = 0.4463\, n_p FA[cat]\sqrt{\frac{n_p FvD}{RT}} \qquad (1)$$

where $\boldsymbol{n_p}$ is number of electrons transferred in the redox event, ***F*** is Faraday constant in C mol^{-1}, ***A*** is electrode area in cm^2, **[*cat*]** is the catalyst concentration in mol cm^{-3}, ***v*** is scan rate in V/s, ***D*** is diffusion coefficient in $cm^2\ s^{-1}$, ***R*** is gas constant in J $K^{-1}\ mol^{-1}$ and ***T*** is temperature in K.

At the steady-state experimental conditions, the scan-rate is independent on the catalytic current (i_{cat}). In this case, i_{cat} could be expressed by following equation

$$i_{cat} = n_{cat}FA[cat]\sqrt{Dk_{cat}} \qquad (2)$$

where $\boldsymbol{n_{cat}}$ is 4 for water oxidation reaction.

Then the TOF value could be calculated according to **equation (3)**:

$$\frac{i_{cat}}{i_p} = 0.359\frac{n_{cat}}{n_p^{3/2}}\sqrt{\frac{k_{cat}}{v}} \qquad (3)$$

Requirements for using method A.[2]

(1) The rate constant of the reaction should be first-order to the concentration of the catalyst.

(2) Redox process should follow Randles–Ševčík equation.

(3) Purely kinetic condition should be achieve (i_{cat} is independent to scan rates).

TOF value is calculated based on three repeated runs and data is listed below.

	Slope	R^2	E_{cat} (*vs* NHE)	TOF (s^{-1})
1	24.101	0.998	1.7	2253
2	25.958	0.980	1.7	2613
3	21.853	0.938	1.7	1852
			TOF	2239±311

Method B: It directly used the **equation (2)** to calculate the TOF (k_{cat}) at a certain concentration of the catalyst and scan rate.[3]In our case (Fig. S15, bottom), i_{cat} was recorded at 1.63 V *vs* NHE with the scan rate of 10 mV s^{-1}, [*cat*] = 0.13 mM.

Method C: If the purely kinetic condition (i_{cat} is independent to scan rates) cannot be reached[2], the TOF value can be estimated at a certain scan rate by using **equation 3** or **4**, which are identical.[4-5]

$$k_{cat} = TOF = 7.759\,\frac{n_p^3}{n_{cat}^2}\left(\frac{i_{cat}}{i_p}\right)^2 v \qquad (4)$$

In our case (Fig. S15, upper), i_{cat} was recorded at 1.7 V *vs* NHE with the scan rate of 100 mV s^{-1}.

Faradaic efficiency (FE)

The oxygen generation was monitored by an Omega PXM409 pressure transducer in a gastight H-shape electrochemical cell, and final oxygen amount was calibrated by gas chromatography (GC2014, Shimadzu). A porous glassy carbon (1 x 1 cm) was used as working electrodes, a Pt mesh was used as the counter electrode and the reference electrode was Ag/AgCl. The **equation 5** was used to calculate the FE.

$$FE = \frac{experimental\ amount\ of\ oxygen}{\frac{Q}{4F}} \qquad (5)$$

Q is the total charge passed during measurement, **F** is Faraday constant in C mol^{-1}.

Kinetic isotope effect (KIE) values calculation from electrochemical experiments

$$pK_{D_2O} = pOD + pD = 14.87$$

$$pK_{H_2O} = pOH + pH = 14$$

The CVS were recorded with a scan rate of 20 mV s^{-1} in 0.1 M phosphate buffer solution:

$pH_{reading}$ = 7.47, *i.e.* PD = 7.87 in D_2O (J. Phys. Chem. B 2019, 123, 8195−8202)

$pH_{reading}$ = 7 in H_2O

The overpotential can be expressed by the following equations in H_2O and D_2O.

$$\eta^{H_2O} = E_{RHE} - 1.229V_{RHE} = E_{Ag/AgCl}^{reading} + 0.059pH + E_{Ag/AgCl}^{H} - 1.229V_{RHE}$$

$$\eta^{D_2O} = E_{RHE} - 1.262V_{RDE} = E_{Ag/AgCl}^{reading} + 0.059pD + E_{Ag/AgCl}^{D} - 1.262V_{RDE}$$

The difference between $E_{Ag/AgCl}^{H}$ and $E_{Ag/AgCl}^{HD}$ is −0.013 V. (Bard, A. Standard potentials in aqueous solution, (Routledge, 2017).)

Therefore, in our case,

$$\eta^{H_2O} - \eta^{D_2O} = 0.059\,\mathrm{x}\,(7 - 7.87) + 0.033 = 0.00533\ \mathrm{V},$$

which is negligible.

Based on **equation (3)**, kinetic deuterium isotope effect was defined as **equation (6)**:

$$KIE = \frac{k_{cat,H_2O}}{k_{cat,D_2O}} = \frac{\left({i_{cat}}/{i_p} \right)^2_{H_2O}}{\left({i_{cat}}/{i_p} \right)^2_{D_2O}} \tag{6}$$

Computational details.

All DFT calculations for the estimation of Gibbs free energies were carried out with the Jaguar 8.3 program package by Schrödinger LLC.[6] Molecular geometries were optimized using Becke's three-parameter hybrid functional and the LYP correlation functional (B3LYP)[7] with D3 correction of Grimme et al.[8-9] with the LACVP** basis set[10]. Frequency calculations were performed on the optimized geometries to verify that the geometries correspond to minima on the potential energy surface. On the basis of the gas-phase optimized geometries, the solvation energies were estimated by single-point calculations using the Poisson−Boltzmann reactive field implemented in Jaguar (PBF) in water. The Gibbs free energy were defined as the following equation G = E(B3LYP-D3/LACVP**) + G_{solv} + ZPE + H_{298} - TS_{298} + 1.9 kcal/mol (the value 1.9 kcal/mol is a concentration correction to the free energy of solvation, which by default is calculated at 1 M (g) to 1 M (aq) in Jaguar).

Single-crystal growth and measurement.

Single crystal of **Ru^{II}-tds** was obtained by slow diffusion of diethyl ether into a 2,2,2-trifluoroethanol solution of **Ru^{II}-tds** at room temperature. The diffraction data of **Ru^{II}-tds** was measured at 293 K by using Mo Kα radiation (λ = 0.71073 Å) on a Bruker D8 Venture single crystal X-ray diffractometer equipped with a kappa geometry goniometer. The dataset was reduced, and absorption correction was applied in APEX3 suite. The crystal structure was solved by direct methods and refined by full-matrix least-squares on F^2 using the SHELX-2018 program package.[11] All non-hydrogen atoms were refined anisotropically defined and hydrogen atoms were placed in calculated positions by means of the "riding" model. A summary of the crystallographic data, the data collection parameters, and the refinement parameters are given in **Table S1**.

Table S1 Summary of the crystallographic data for **Ru-tds**

	**Ru^{II}(tds-*κ*-N^{3}O)Py$_2$** (CCDC NO. 2209277)	**Ru^{II}(tds-*κ*-N^{3}O^2)Py$_2$** (CCDC NO. 2209276)
Empirical formula	$C_{29}H_{25}F_6N_5O_8RuS_2$	$C_{29}H_{25}F_6N_5O_8RuS_2$
Formula weight	850.73	850.73
Crystal system	Orthorhombic	Monoclinic
Space group	Pca2(1)	C2/c
a/Å	24.426(5)	17.798(4)
b/Å	10.129(2)	9.952(2)
c/Å	13.528(3)	18.278(4)
α	90	90
β	90	93.75(3)
γ	90	90
V/Å^3	3347.0(12)	3230.4(11)
Z	4	4
T/K	293	293
λ/Å	0.71073	0.71073
F(000)	1712	1712
ρ_{calcd}/g cm^{-3}	1.688	1.749
μ/mm^{-1}	0.684	0.708
Measured refls.	112207	75434
Independent refls.	7139	4062
No. of parameters	462	252
R_{int}	0.1976	0.1785
$^a R$ indices [$I > 2\sigma(I)$]; R_1, wR_2	0.0626, 0.1569	0.0650, 0.1640
GOF	1.063	1.138

^{a} $R_1 = \sum\|F_o|-|F_c\|/\sum|F_o|$, $wR_2 = [\sum w(F_o^2-F_c^2)^2/\sum w(F_o^2)^2]^{1/2}$

Synthesis of the ligand [2,2':6',2''-terpyridine]-6,6''-disulfonic acid (H_2tds).

A mixture of 6,6′′-dibromo-2,2′:6′,2′′-terpyridine (1.0 g, 2.56 mmol) and sodium hydrosulfide (1.5 g, 26 mmol) in DMF was heated at 140 °C over 24 hours under N_2. The greenish powder was obtained by removing the solvent under vacuum. Dissolving the green powder in 70 mL water, and acetic acid was added drop wise into the solution. The product ([2,2':6',2''-terpyridine]-6,6''-dithiol) was obtained as a yellow powder after filtration and washing with water. The obtained [2,2':6',2''-terpyridine]-6,6''-dithiol was then dissolved in 50 mL nitric acid (70%) and heated at 100 °C for 2 h, followed by removing the solvent under vacuum. The product of [2,2':6',2''-terpyridine]-6,6''-disulfonic acid (**H_2tds**) was obtained as a light yellow powder (yield: 510 mg, 51%). ^{1}H NMR (500 MHz, D_2O) δ 8.71 (dd, J = 8.6, 7.4 Hz, 1H), 8.62 (d, J = 7.9 Hz, 2H), 8.40 (d, J = 7.9 Hz, 2H), 8.25 (t, J = 7.9 Hz, 2H), 8.11 (d, J = 7.7 Hz, 2H). ^{13}C NMR (126 MHz, D_2O) δ 159.29, 148.35, 147.69, 146.52, 141.00, 125.78, 125.53, 123.59.

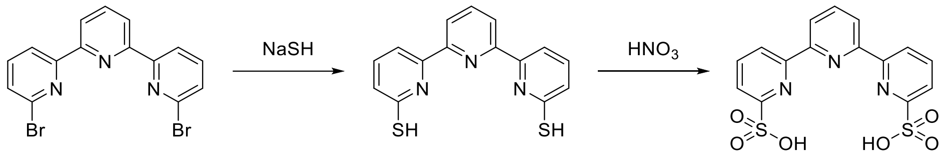

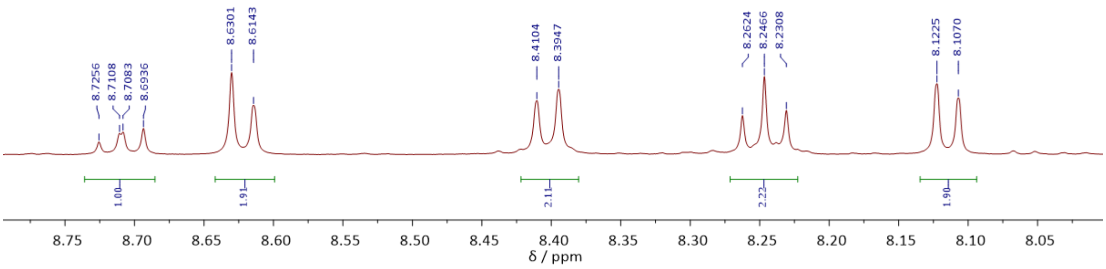

Figure S1 ^{1}H NMR spectrum of **H_2tds** in D_2O.

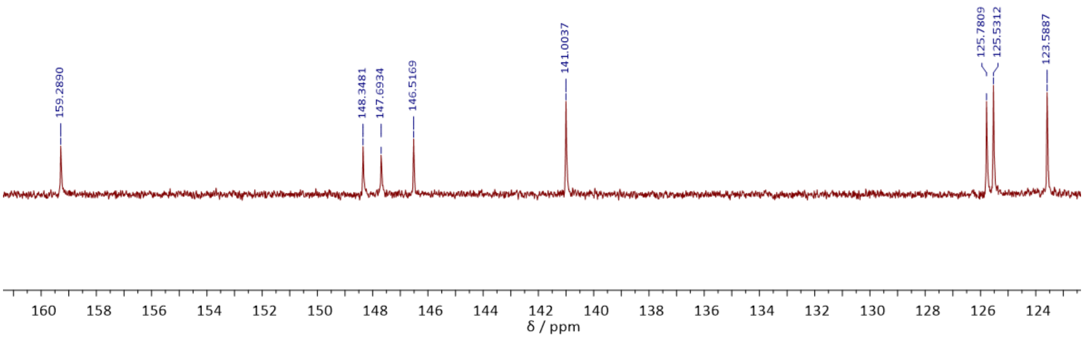

Figure S2 ^{13}C NMR spectrum of **H_2tds** in D_2O.

Synthesis of the catalyst Ru-tds.

A mixture of **H_2tds** (136 mg, 0.35 mmol), $Ru(DMSO)_4Cl_2$ (168 mg, 0.35 mmol) and 0.3 mL of triethylamine in ethanol (10 mL) was heated at 80 °C under N_2, and 5 mL of pyridine was added after the above mixture turning reddish. The crude product was purified by column chromatography (SiO_2, dichloromethane/methanol 1:5) to afford 30 mg of **Ru-tds** as a dark-red powder (yield: 30 mg, 13%). ^{1}H NMR (500 MHz, D_2O/CD_3OD 1/3) δ 8.66 (d, J = 8.2 Hz, 2H), 8.57 (d, J = 8.1 Hz, 2H), 8.27 (d, J = 6.9 Hz, 2H), 8.19 (t, J = 7.9 Hz, 2H), 8.16 – 8.09 (m, 5H), 7.62 (t, J = 7.7 Hz, 2H), 7.14 – 7.08 (t, 7.4 Hz, 4H). ^{1}H NMR (500 MHz, DMSO-*d*$_6$) δ 8.78 (d, J = 8.1 Hz, 2H), 8.67 (d, J = 7.2 Hz, 2H), 8.13 (m, J = 10.5, 4.1 Hz, 9H), 7.59 (t, J = 7.4 Hz, 2H), 7.09 (t, J = 6.8 Hz, 4H). ^{13}C NMR (126 MHz, DMSO-*d*$_6$) δ 165.25, 161.34, 157.53, 152.59, 138.07, 136.86, 133.02, 124.78, 124.73, 124.38, 124.32. HR-MS (calcd for $C_{26}H_{19}N_5O_6Ru(II)S_2{}^+$ [**Ru-tds** + H^+]$^+$: 651.9904; found: 651.9896).

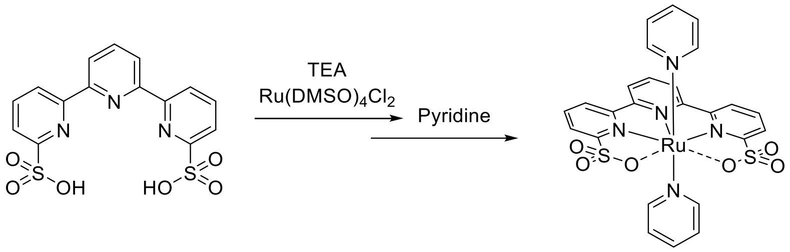

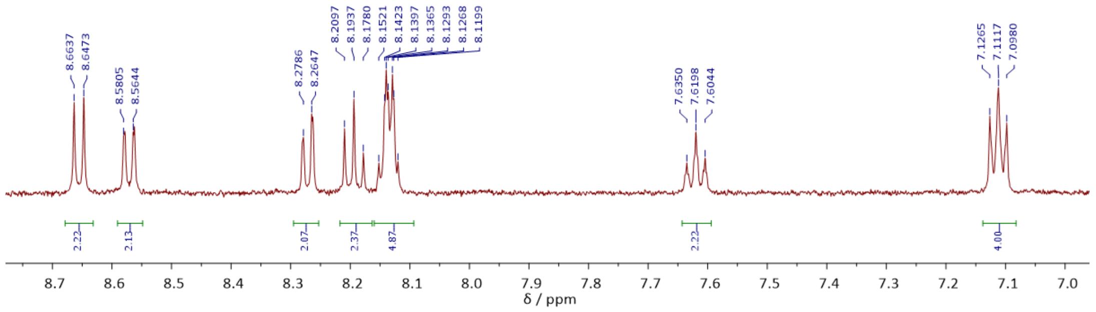

Figure S3 ^{1}H NMR spectrum of **Ru-tds** in D_2O/CD_3OD (1/3).

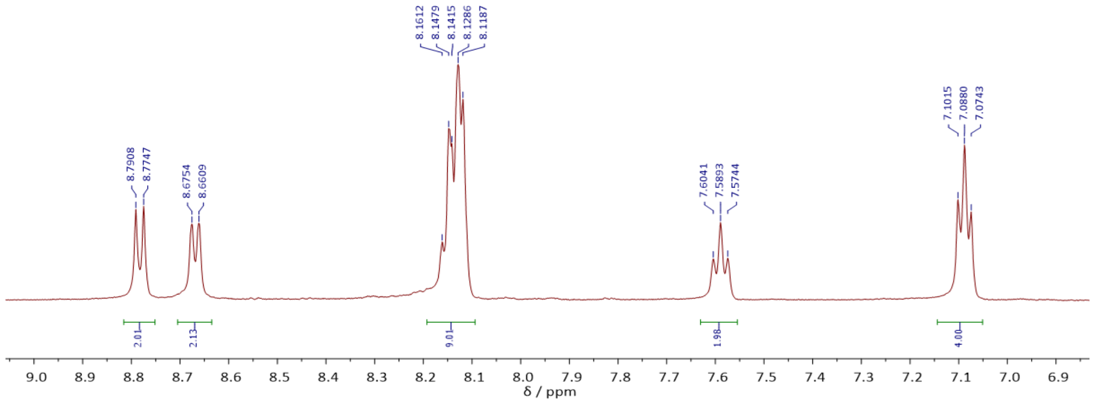

Figure S4 ^{1}H NMR spectrum of **Ru-tds** in DMSO-*d*$_6$.

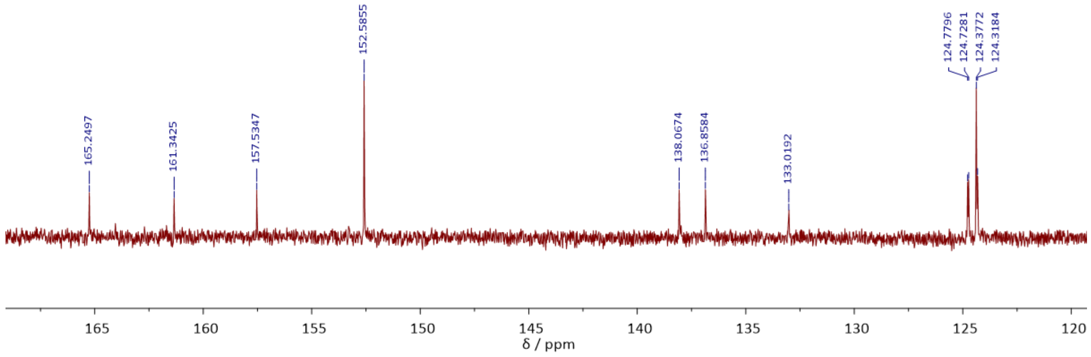

Figure S5 ^{13}C NMR spectrum of **Ru-tds** in DMSO-d_6.

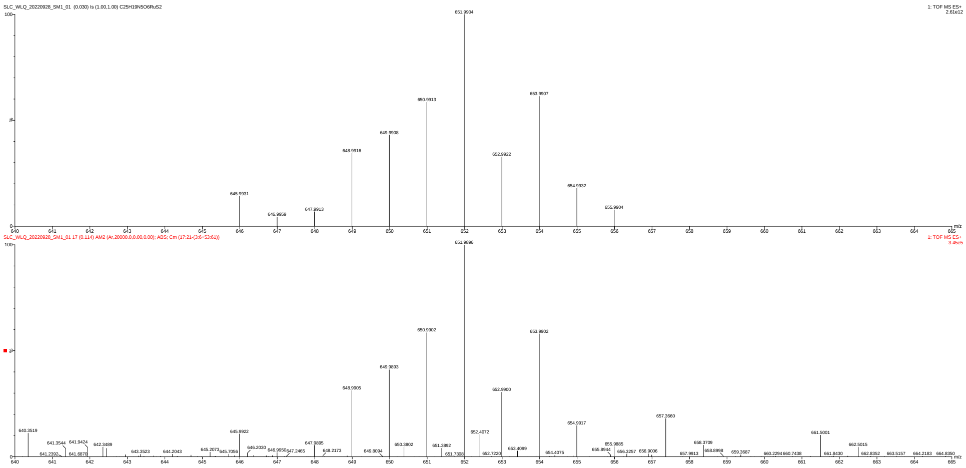

Figure S6 HRMS of **Ru-tds** (bottom) and calculated mass spectrum (upper).

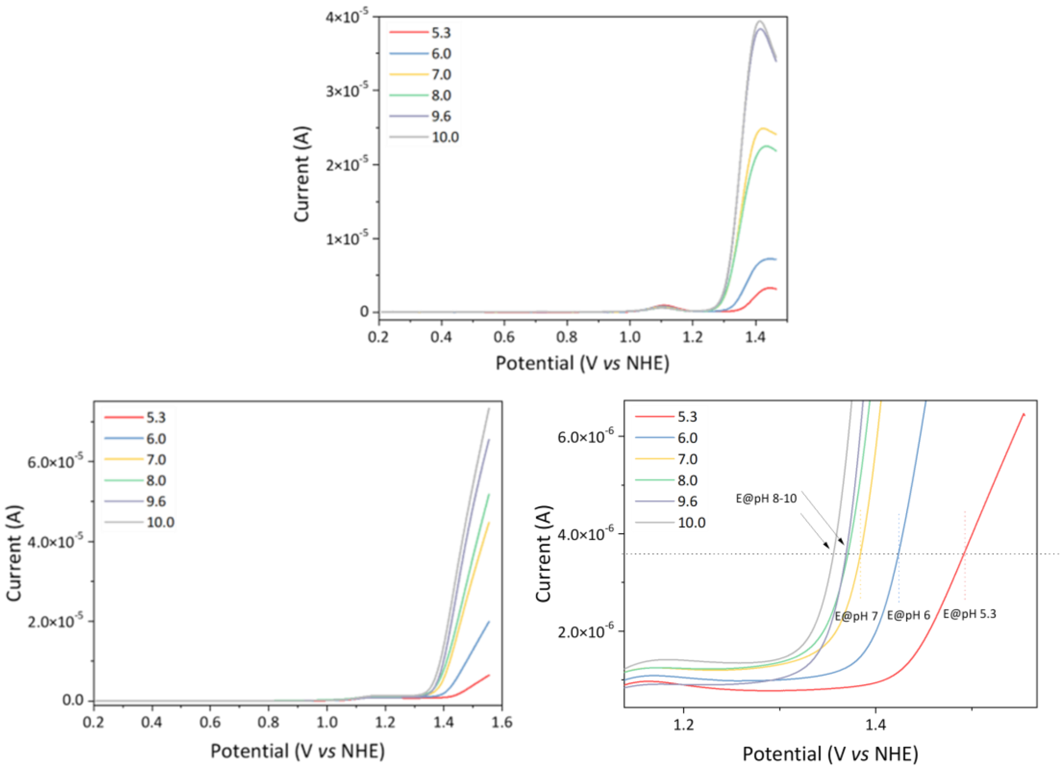

Figure S7 DPVs (upper) and LSVs (bottom, scan rate = 100 mV s^{-1}) without background-subtraction of 0.13 mM **Ru-tds** under various pH conditions, working electrode: BDD.

Note: The redox process of RuV/IV aqua species might be overlapped with the catalytic current, and we cannot observe redox signals from CV and LSV. Instead, we estimated the potential of RuV/IV aqua species by extracting data from their LSVs at the same currents (ca. 3.7 μA , the values shown in Fig. 3b and S7 right bottom). For the pH invariance signals around 1.45 V in DPVs (Fig. S7 upper), they are unlikely responsible for the oxidation of Ru-aqua (V/IV) species because one cannot form the high valent Ru(O)/Ru(OH) species without PCET steps. Therefore processes accounting for those signals are unclear and might be from the oxidation of non-aqua species of Ru(V/IV). The inclusion of those signals in Figure 3b, in our opinion, would give more misleading information than rewarding. Thus we only used the data extracted from the catalytic currents (LSV) instead of DPV to construct the potential *vs.* pH diagram.

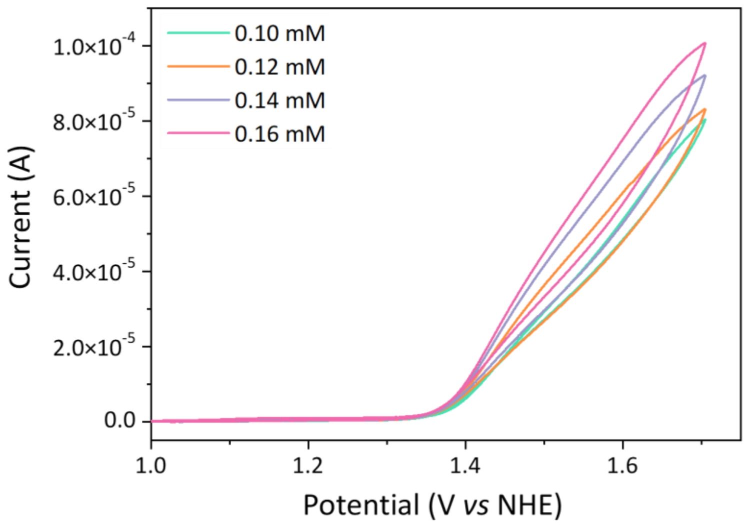

Figure S8 CVs without background-subtraction for different concentrations of **Ru-tds** in a 0.1 M phosphate buffer solution containing 1% CF3CH2OH, pH = 7, scan rate = 20 mV s^{-1}, working electrode: BDD.

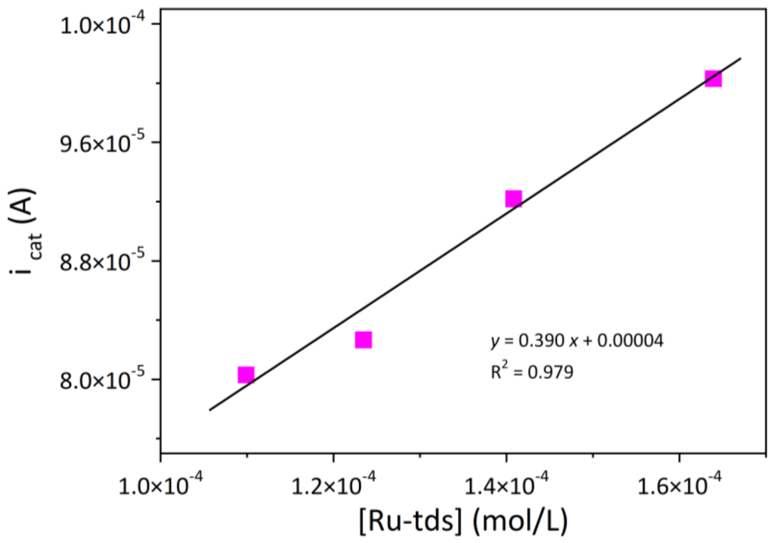

Figure S9 Plot of the i_{cat} at 1.70 V versus [**Ru-tds**].

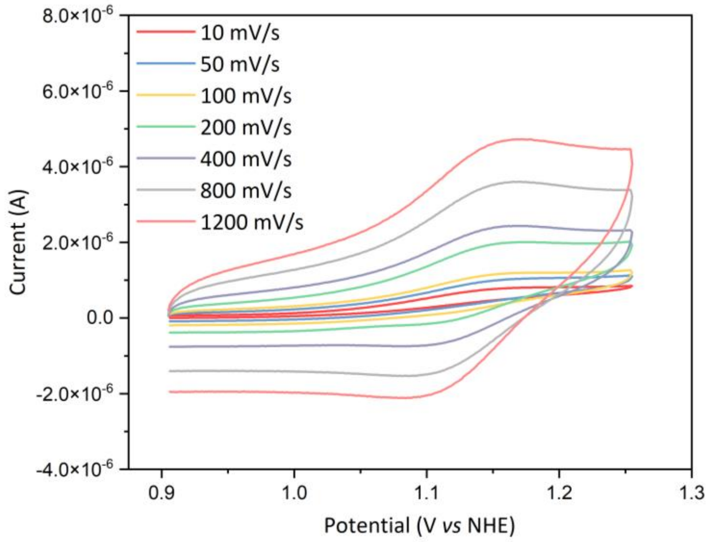

Figure S10 CVs without background-subtraction of 0.13 mM **Ru-tds** at different scan rates in a 0.1 M phosphate buffer solution containing 1% CF_3CH_2OH, pH = 7, working electrode: BDD.

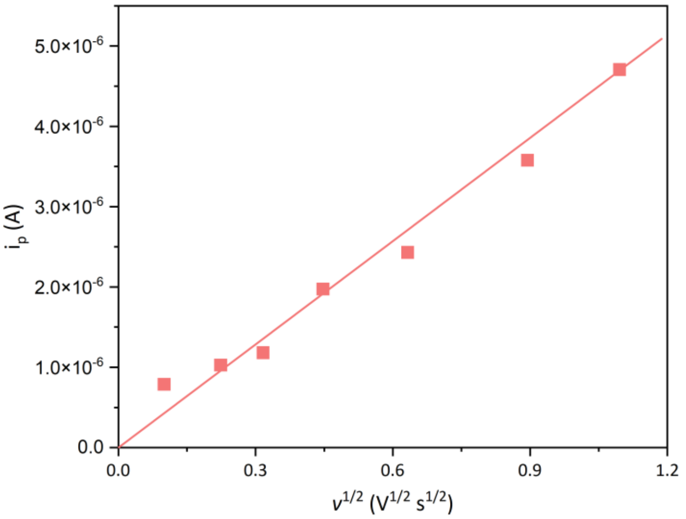

Figure S11 Plot of the peak current ($Ru^{IV/II}$ couple) for **Ru-tds** *vs*. the square root of scan rate.

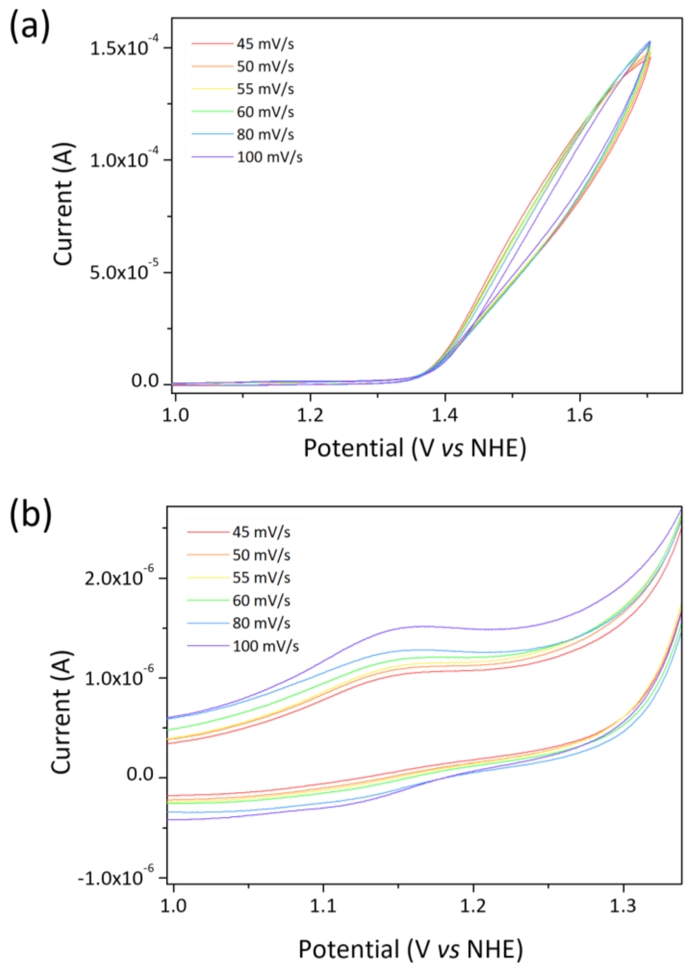

Figure S12 (a) CVs without background-subtraction of 0.13 mM **Ru-tds** at different scan rates in a 0.1 M phosphate buffer solution containing 1% CF_3CH_2OH, pH = 7, working electrode: BDD.(b) Enlargement of the 1.0−1.35 V range

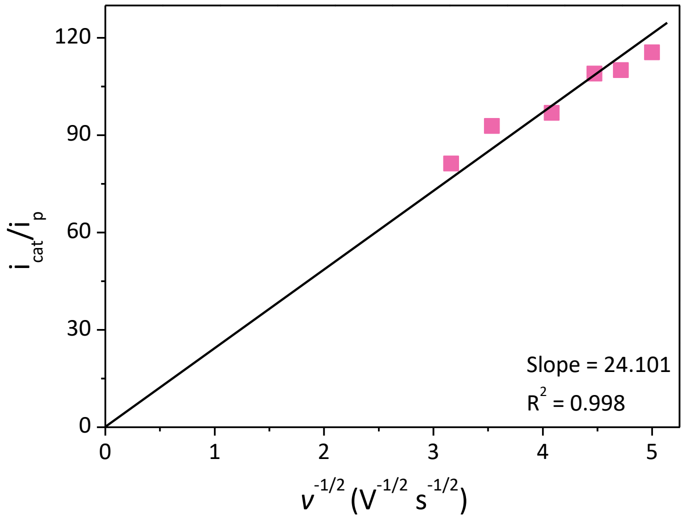

Figure S13 Plot of the i_{cat}/i_p for **Ru-tds** *vs.* $v^{-1/2}$, i_{cat} measured at 1.7 V.

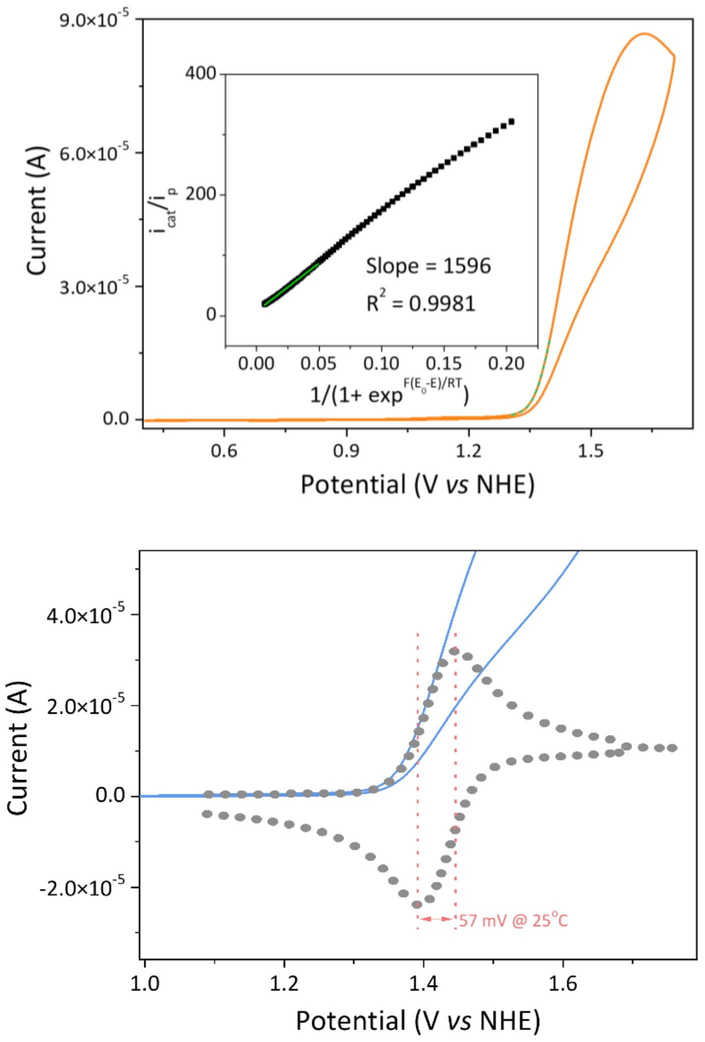

Figure S14 . Upper: CV without background-subtraction of 0.13 mM **Ru-tds** at pH 7.0 in a 0.1 M phosphate buffer solution containing 1% CF_3CH_2OH, scan rate = 10 mV s^{-1}, working electrode: BDD. Inset: FOWA plot of the catalytic current. The blue dashed line represents the data used for calculation of the TOF_{max}. Bottom: the method used to estimate the E_o for FOWA.

Note 1: We assumed an ideal one electron transfer process for Ru(V/IV) aqua species (grey dot, duck shape), and a scenario where the catalytic onset potential overlapped with the redox onset signal as shown in the bottom of Fig S14. At room temperature, the peak to peak separation should be around 57 mV.[12] Then the estimated E_0 is around 1.41 V.

Note 2: We also used the E_0 estimated based on Appel and Helm method ($E_{cat/2}$) [1] to calculated the TOF_{max}, and the obtained value is similar ($TOF_{max} \approx 14000\ s^{-1}$).

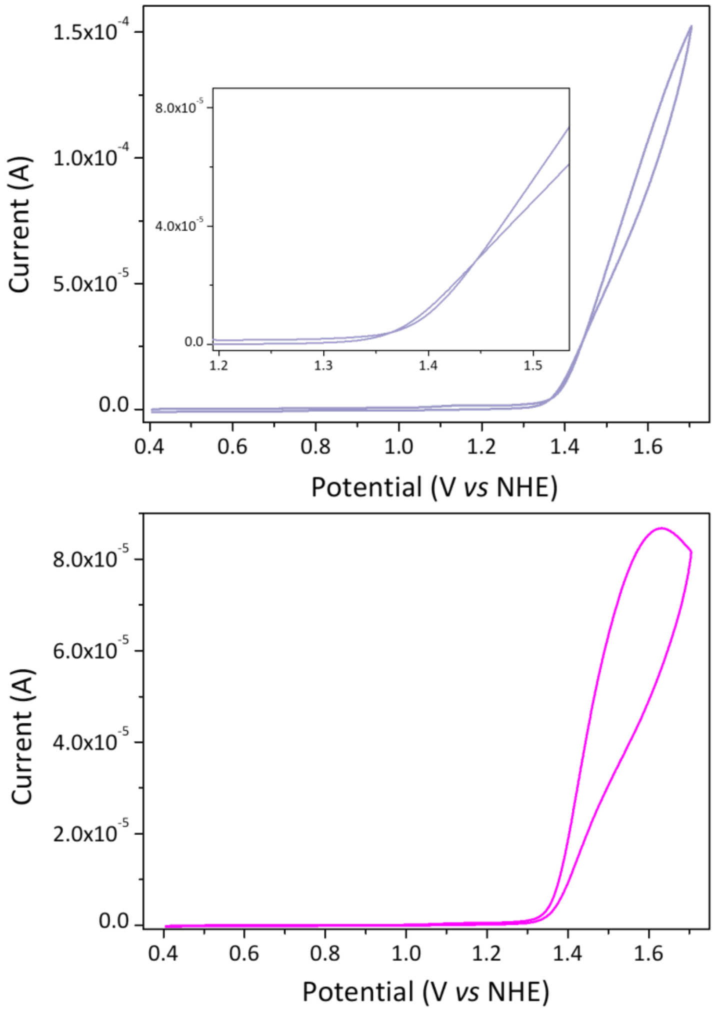

Figure S15 CV without background-subtraction of 0.13 mM **Ru-tds** at pH 7.0 in a 0.1 M phosphate buffer solution containing 1% CF_3CH_2OH, scan rate = 100 mV s^{-1} (upper) and 10 mV s^{-1} (bottom) , working electrode: BDD.

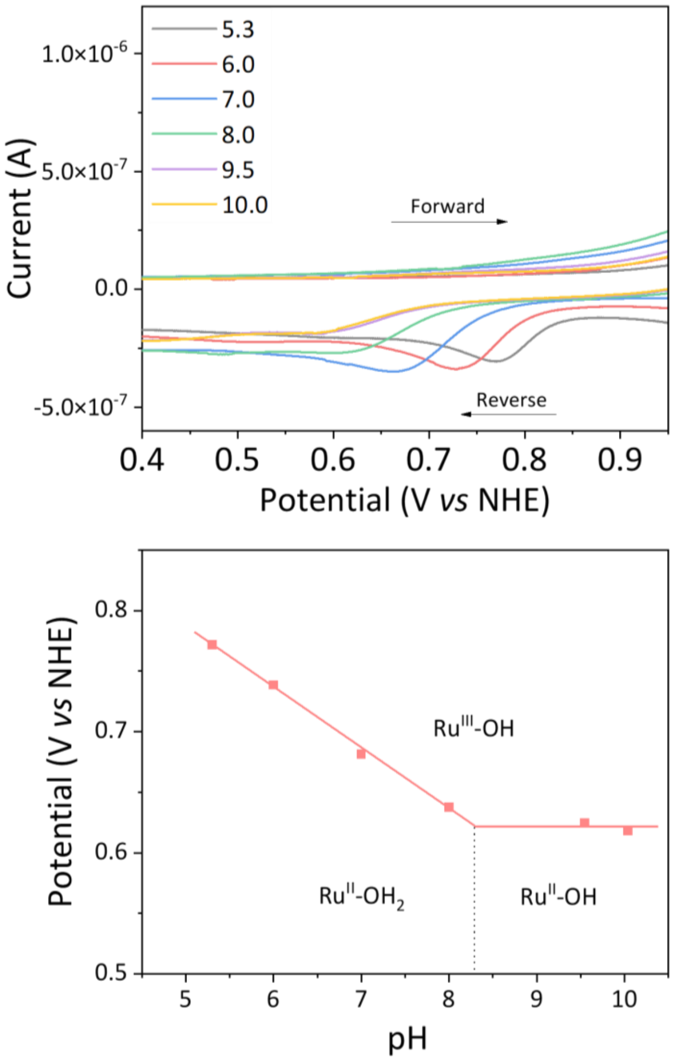

Figure S16 CVs without background-subtraction of 0.13 mM **Ru-tds** under various pH conditions (upper) and the pH *vs.* potential diagram (extracted from reverse scan, bottom), scan rate = 100 mV s^{-1}, working electrode: BDD.

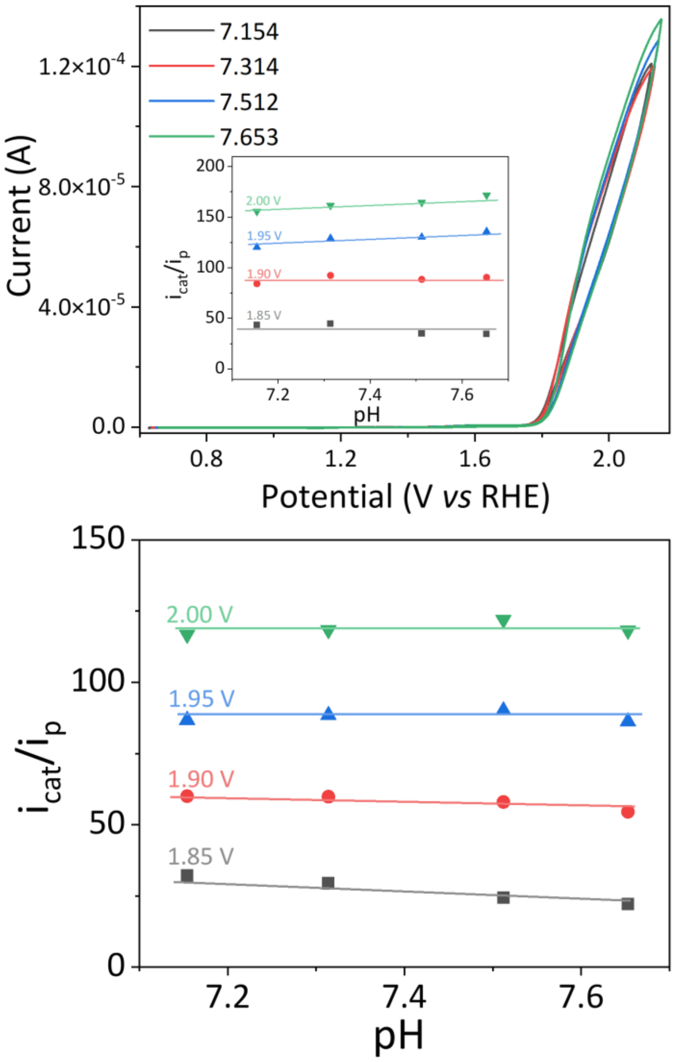

Figure S17. Upper: CVs without background-subtraction of 0.13 mM **Ru-tds** in a 0.1 M phosphate buffer solution in various pH, working electrode: BDD. Inset: forwards scan catalytic current *vs.* pH. Bottom: backwards scan catalytic current *vs.* pH.

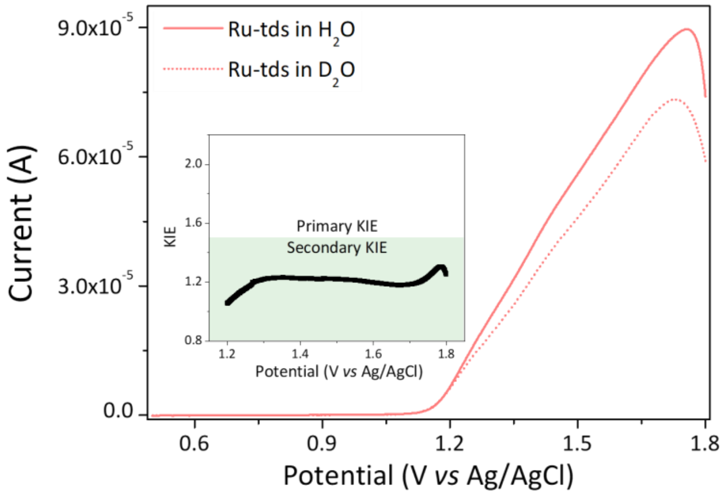

Figure S18. (a) Backward scans LSV without background-subtraction of 0.13 mM **Ru-tds** in a 0.1 M phosphate buffer solution in (H_2O and D_2O, pH = 7 and pD = 7.87) containing 1% CF_3CH_2OH, scan rate = 10 mV s^{-1}, working electrode: BDD.

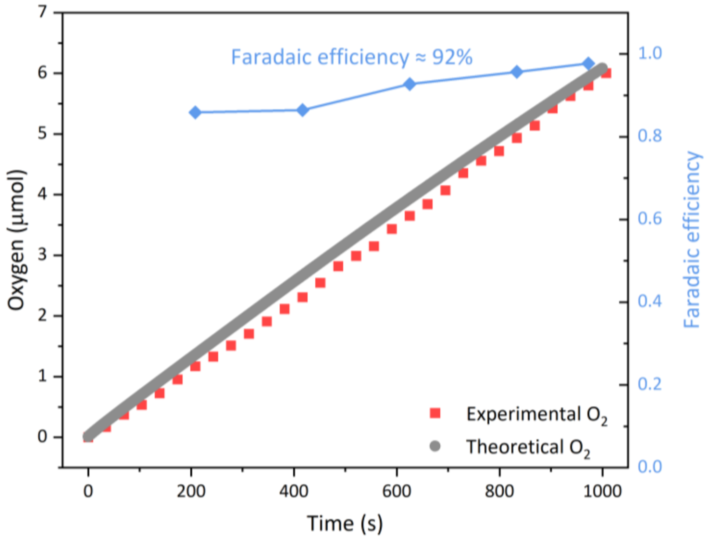

Figure S19. Faradaic efficiencies of **Ru-tds** (10 mM) for water oxidation in a 0.1 M phosphate buffer solution containing 1% CF_3CH_2OH, working electrode: porous glassy carbon (1 x 1 cm).

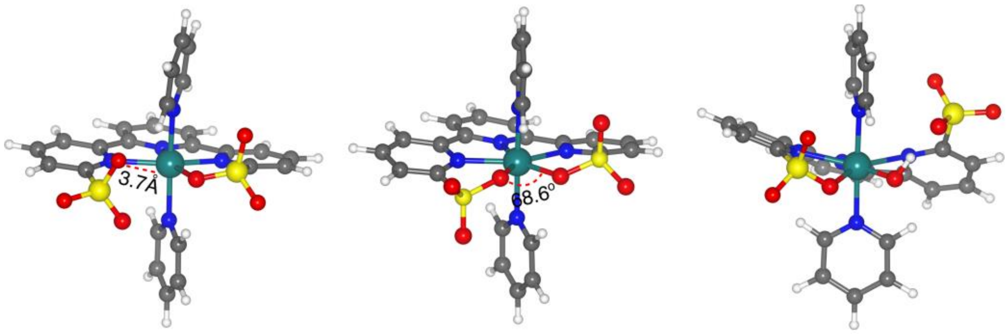

(1) (2) (3)

Figure S20. The optimized geometries of Ru^{II} (1), Ru^{IV} (2) and Ru^{IV}-OH (3) species.

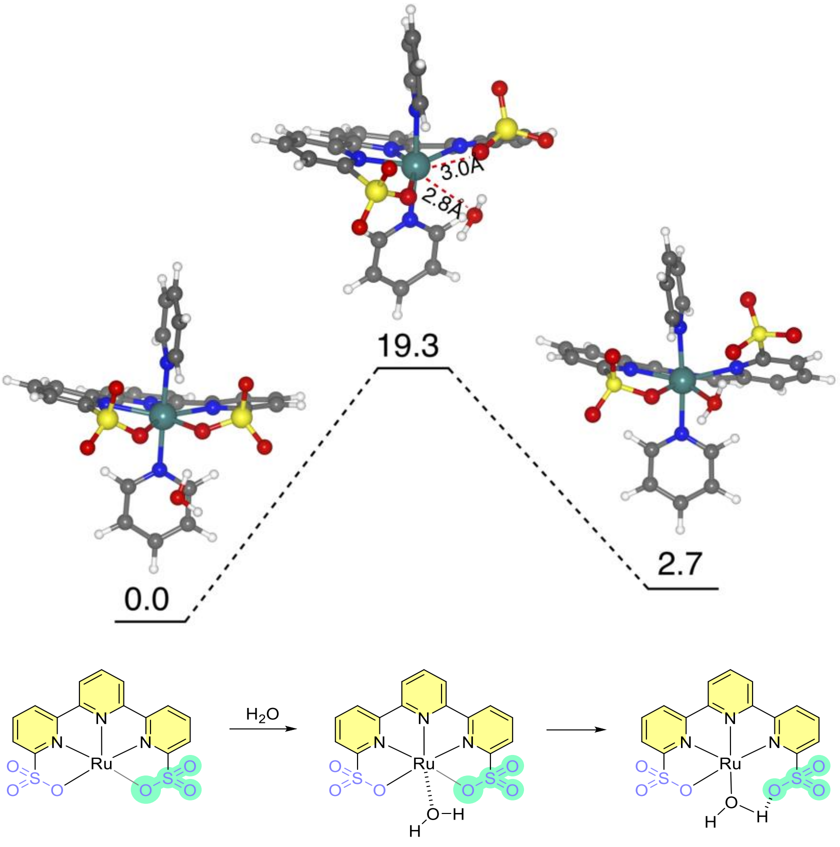

Figure S21. The energy profiles of ligand exchange on Ru^{IV} at pH 7.0. The units of energies are kcal mol^{-1}.

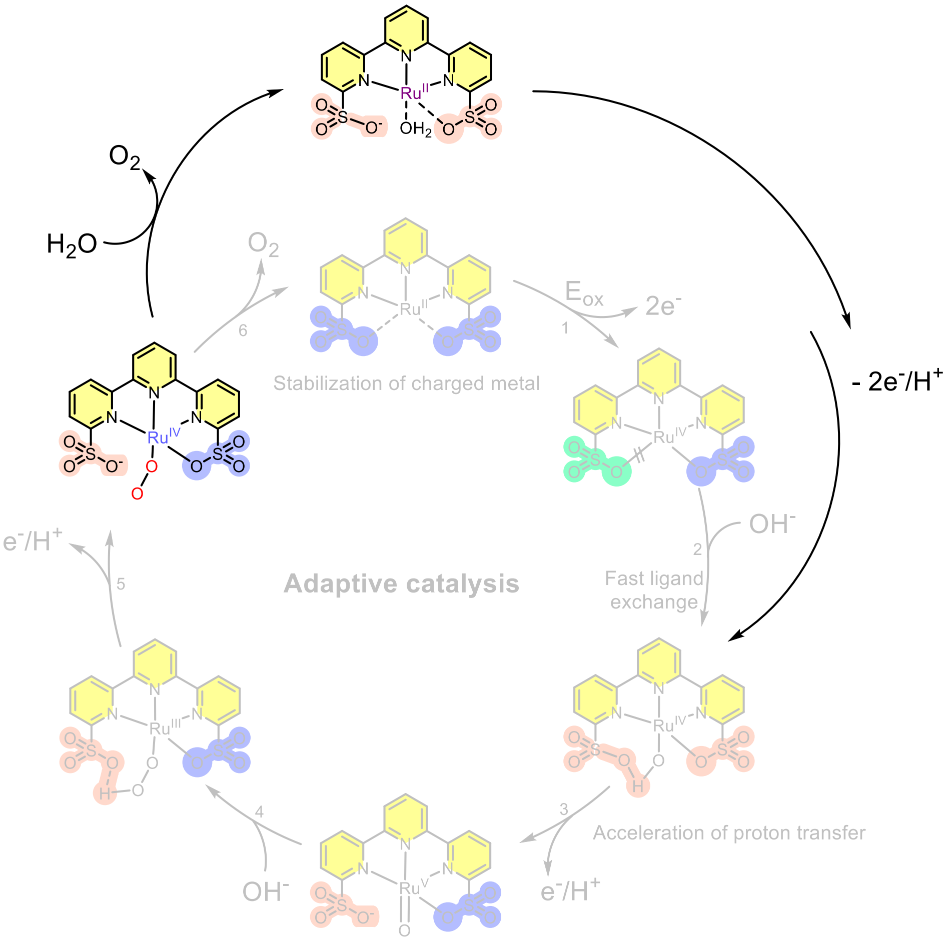

Scheme S1. Possible alternative pathway to complete the catalytic cycle.

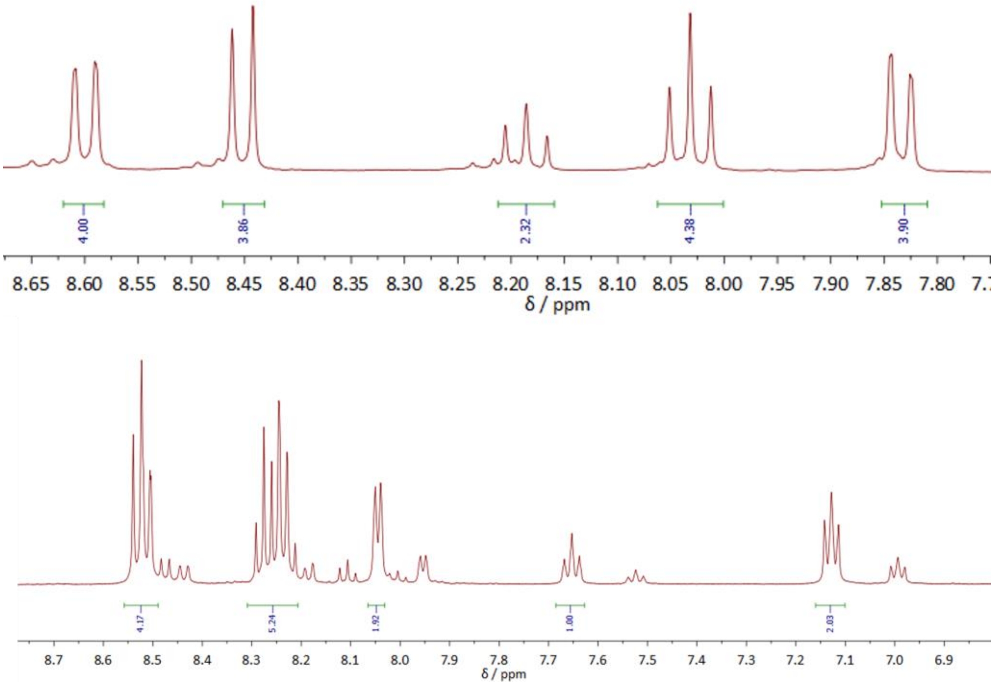

Figure S22 ^{1}H NMR spectra of $Ru(tds)_2$ (upper) and Ru(tds)(py)(DMSO) (bottom).

Table S2 Comparison of water oxidation activity of **Ru-tds** with other Ru-based catalysts under pH neutral conditions.

Structure	Reference	Method	$E_{onset}-E_{O_2/H_2O}$ (mV)	TOF (s^{-1})	TOF_{max} (s^{-1})
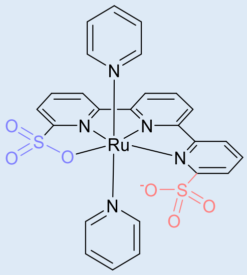 Ru-tds	This work	A	530	2239 ±311 s^{-1}	/
	This work	B	530	3242	/
	This work	C	530	4195	/
	This work	FOWA	530	/	12000
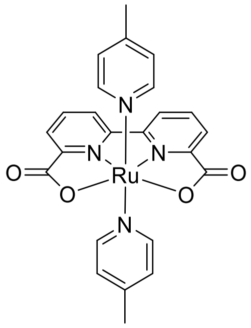 Ru-bda	Nat Commun, 2021, 12, 373.<br>J. Am. Chem. Soc. 2009, 131, 1039	C	320	300	/
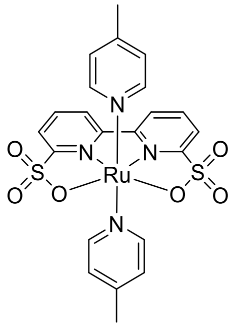 Ru-bds	Nat Commun, 2021, 12, 373.	C	420	1290<br>0	/

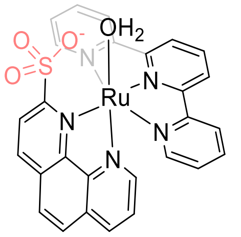<br>Grotjahn-Cat	Angew. Chem. Int. Ed. 2021, 60, 1540.	B	800	2595	/
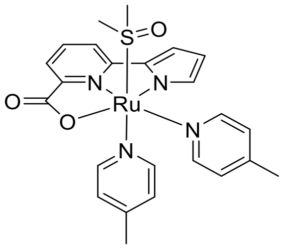	Inorg. Chem. 2021, 60, 15627	C	352	16.34	/
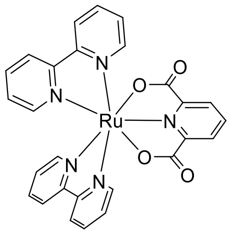	ChemSusChem 2019, 12, 1949	FOWA	653	/	3400
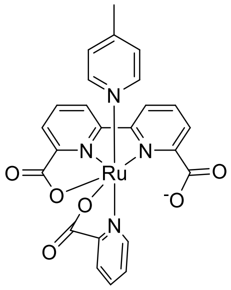	Inorg. Chem. 2020, 59, 4443	FOWA	500	/	0.7
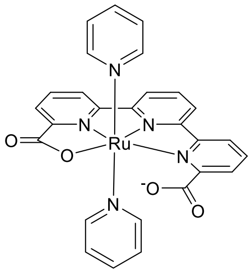<br>Ru-tda	J. Am. Chem. Soc. 2015, 137, 10786	FOWA	470-570	/	8000
Ru-tpa	J. Am. Chem. Soc. 2020, 142, 5068<br>*ACS Catal.* 2021, 11, 5240<br>J. Am. Chem. Soc. 2017, 139, 15347	FOWA	530	/	16000

Structure evolution of Ru-tpa

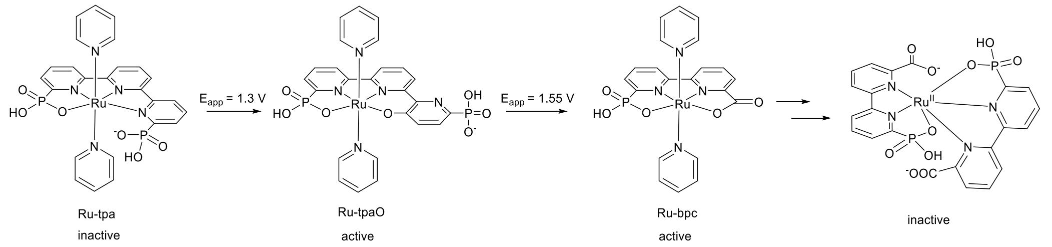

References

1. Appel, A. M.; Helm, M. L., Determining the Overpotential for a Molecular Electrocatalyst. *ACS Catal.* **2014,** *4* (2), 630-633.

2. Okamura, M.; Kondo, M.; Kuga, R.; Kurashige, Y.; Yanai, T.; Hayami, S.; Praneeth, V. K.; Yoshida, M.; Yoneda, K.; Kawata, S., A pentanuclear iron catalyst designed for water oxidation. *Nature* **2016,** *530* (7591), 465-468.

3. Nash, A. G.; Breyer, C. J.; Vincenzini, B. D.; Elliott, G. I.; Niklas, J.; Poluektov, O. G.; Rheingold, A. L.; Smith, D. K.; Musaev, D. G.; Grotjahn, D. B., An Active-Site Sulfonate Group Creates a Fast Water Oxidation Electrocatalyst That Exhibits High Activity in Acid. *Angew. Chem. Int. Ed.* **2021,** *60* (3), 1540-1545.

4. Yang, J.; Wang, L.; Zhan, S.; Zou, H.; Chen, H.; Ahlquist, M. S. G.; Duan, L.; Sun, L., From Ru-bda to Ru-bds: a step forward to highly efficient molecular water oxidation electrocatalysts under acidic and neutral conditions. *Nat. Commun.* **2021,** *12* (1), 373.

5. Ngo, K. T.; McKinnon, M.; Mahanti, B.; Narayanan, R.; Grills, D. C.; Ertem, M. Z.; Rochford, J., Turning on the Protonation-First Pathway for Electrocatalytic CO2 Reduction by Manganese Bipyridyl Tricarbonyl Complexes. *J. Am. Chem. Soc.* **2017,** *139* (7), 2604-2618.

6. Bochevarov, A. D.; Harder, E.; Hughes, T. F.; Greenwood, J. R.; Braden, D. A.; Philipp, D. M.; Rinaldo, D.; Halls, M. D.; Zhang, J.; Friesner, R. A., Jaguar: A high-performance quantum chemistry software program with strengths in life and materials sciences. *Int. J. Quantum Chem.* **2013,** *113* (18), 2110-2142.

7. Zhao, Y.; Truhlar, D. G., The M06 suite of density functionals for main group thermochemistry, thermochemical kinetics, noncovalent interactions, excited states, and transition elements: two new functionals and systematic testing of four M06-class functionals and 12 other functionals. *Theor. Chem. Acc.* **2008,** *120* (1), 215-241.

8. Grimme, S.; Antony, J.; Ehrlich, S.; Krieg, H., A consistent and accurate ab initio parametrization of density functional dispersion correction (DFT-D) for the 94 elements H-Pu. *J. Chem.Phys.* **2010,** *132* (15), 154104.

9. Goerigk, L.; Grimme, S., A thorough benchmark of density functional methods for general main group thermochemistry, kinetics, and noncovalent interactions. *Phys. Chem. Chem.Phys.* **2011,** *13* (14), 6670-6688.

10. Hay, P. J.; Wadt, W. R., Ab initio effective core potentials for molecular calculations. Potentials for K to Au including the outermost core orbitals. *J. Chem.Phys.* **1985,** *82* (1), 299-310.

11. Sheldrick, G. M., SHELXT–Integrated space-group and crystal-structure determination. *Acta Crystallogr. A* **2015,** *71* (1), 3-8.

12. Elgrishi, N.; Rountree, K. J.; McCarthy, B. D.; Rountree, E. S.; Eisenhart, T. T.; Dempsey, J. L., A Practical Beginner's Guide to Cyclic Voltammetry. *J. Chem. Educ.* **2018,** *95* (2), 197-206.

Cartesian Coordinates in Å and Energies of the Calculated Geometries.

1

E (B3LYP-D3/LACVP**)(a.u.) = -2579.546470

ZPE (kcal mol^{-1}) = 263.844

G_{solv} (kcal mol^{-1})= -46.816

ΔH_{298} (kcal mol^{-1})= 20.242

ΔS_{298} (cal K^{-1} mol^{-1})= 189.819

Cartesian coordinates

Atom	x	y	z
Ru39	9.2835262261	5.2813039660	4.3292533271
S40	7.9353602490	7.1605165824	7.2126850508
S41	12.3369715514	6.0932961887	4.7569404986
N42	9.3664200211	6.9953686645	3.0739506470
N43	10.8036167393	4.4531532186	3.3000721961
O44	10.9734197964	6.2003014775	5.4325476396
N45	7.2366631057	5.7650432477	4.9221753355
C46	13.1087965265	4.0785656814	2.8702570767
H47	14.1405218054	4.3060915594	3.1127871245
O48	13.3857725600	5.6070779226	5.6613485412
O49	12.6418928957	7.2484459966	3.8981944362
C50	12.7586278956	3.1420099994	1.8956767671
H51	13.5312096879	2.6119411661	1.3474225258
N52	9.3471852753	3.6625204659	5.7287608881
N53	8.2547383696	4.2484427269	3.0287995138
C56	6.7677881273	6.4872993891	5.9510704835
C57	11.4122234255	2.8879016473	1.6130472083
H58	11.1378073605	2.1736114366	0.8447122029
C59	6.9022764728	4.3427917709	2.9888563972
C60	10.4285260133	3.5624232341	2.3349059279
C61	5.4006772090	6.7391764267	6.1207057740
H62	5.1042252498	7.3345311951	6.9758148446
O63	7.0203457033	7.6914688661	8.2444404281
C64	6.3385369172	5.2123061052	4.0421651345
C65	8.2844284899	2.7163363740	1.1993502273
H66	8.8311404221	2.0891250864	0.5044998571
C67	12.0835183445	4.7313378529	3.5494031581
C69	6.8903947871	2.7956754781	1.1392390943
H70	6.3484329207	2.2216801081	0.3949268390
C71	10.1450372965	3.7704784518	6.8120657266
H72	10.7018980005	4.6924441130	6.9010473680
C74	8.9657029778	3.4621255393	2.1584786643
C75	4.4934286396	6.2203893170	5.2086973655

H76	3.4292551575	6.4097186029	5.3133047726
C77	6.1927438734	3.6116892578	2.0307778894
H78	5.1120556908	3.6705494539	1.9831878115
C79	4.9677288957	5.4339929614	4.1604965094
H80	4.2784820168	4.9970767511	3.4470784964
C81	9.6476695610	8.1831207473	3.6501074222
H82	9.6996699673	8.1874299976	4.7335570720
C83	8.5954635712	2.5506495546	5.5904778664
H84	7.9556525903	2.5019911789	4.7180995792
O85	8.6874048479	8.1891605700	6.4541948361
O89	8.7083686688	5.9604360427	7.6073190561
C90	8.6166113609	1.5108992137	6.5094318941
H91	7.9869679645	0.6416444017	6.3500336476
C92	9.7428024525	9.2646348945	1.5026322376
H93	9.8951164030	10.1483280175	0.8898871432
C95	10.2236563830	2.7625160074	7.7683975885
H96	10.8799305310	2.9005575077	8.6203649358
C97	9.8362442342	9.3334127972	2.8882019078
H98	10.0641824529	10.2635231596	3.3964744377
C99	9.2750569303	6.9268262061	1.7287725391
H100	9.0565100477	5.9543382586	1.3036628860
C101	9.4533336262	8.0329252219	0.9117932894
H102	9.3723336060	7.9239788072	-0.1648676184
C103	9.4511883124	1.6150425474	7.6228554964
H104	9.4910136652	0.8198055438	8.3614123884

2

E (B3LYP-D3/LACVP**)(a.u.) = -2578.972724

ZPE (kcal mol^{-1}) = 264.679

G_{solv} (kcal mol^{-1})= -153.034

ΔH_{298} (kcal mol^{-1})= 20.606

ΔS_{298} (cal K^{-1} mol^{-1})= 200.405

Cartesian coordinates

Atom	x	y	z
Ru1	8.0034972335	5.6150956935	13.6770984615
S2	6.4855218315	7.3890182985	11.4990486119
S3	9.5155415007	7.3942420584	15.8584111486
N4	7.3181673915	4.9193138528	11.7157133595
N5	8.6821978584	4.9245567700	15.6413262021
N6	5.9965883467	5.6850948225	14.4215276355
N7	10.0120746151	5.6839826565	12.9368157540
N8	8.0043401651	3.4611120648	13.6782971023

C9	7.8115282556	2.7732703281	12.5224289030
C10	8.1983329012	2.7756970352	14.8352986644
C11	6.8345661215	5.7654557069	10.8057069459
C12	9.1597824444	5.7727325197	16.5526397094
C13	6.5643341029	5.4068723654	9.4914799335
C14	9.4242837827	5.4164025593	17.8686546014
H15	6.1870968183	6.1554311844	8.8020057253
C16	7.8269132377	1.3776481486	12.4937378684
C17	8.1888440318	1.3800739940	14.8657282597
H18	7.6754363022	0.8518783082	11.5590988928
H19	8.3416292516	0.8561419174	15.8011757359
C20	8.0097453848	0.6734797735	13.6801995531
C21	7.4837827022	3.6162079172	11.3808183350
C22	8.5193516760	3.6211447680	15.9768688502
O23	5.1591804645	7.3002606446	12.0919466313
O24	10.8477976842	7.3018258864	15.2793395633
C25	5.1154349265	4.6914764570	14.1714768443
C26	10.8876731114	4.6818558226	13.1725542719
H27	5.4907773278	3.8220525961	13.6472432501
H28	10.5072330393	3.8060975164	13.6823035970
C29	5.5620798751	6.8008596917	15.0462464887
C30	10.4532991052	6.8069204528	12.3299991872
H31	6.2901099674	7.5857808138	15.1988574070
H32	9.7296889155	7.5980045236	12.1885700254
C33	6.8087522949	4.0875490003	9.1150663221
C34	9.1816240758	4.0969790792	18.2455326082
H35	6.6376535531	3.7640570227	8.0932630872
C36	7.2520636559	3.1775419744	10.0768533616
C37	8.7462816240	3.1845864634	17.2823709118
H38	7.4123385142	2.1395818730	9.8121775946
H39	8.5884116698	2.1463327335	17.5472873889
C40	4.2421260981	6.9429762823	15.4570389497
C41	11.7742799024	6.9478036475	11.9219454102
H42	3.9398487157	7.8590863428	15.9523069186
H43	12.0819611311	7.8698315236	11.4412025238
C44	3.7844691462	4.7701340813	14.5498218431
C45	12.2191829845	4.7587545256	12.7958643164
H46	3.1133805704	3.9486269966	14.3239583380
H47	12.8854058045	3.9299560197	13.0091187389
C48	3.3345572747	5.9173863153	15.2065738521
C49	12.6758359258	5.9132297998	12.1566286344
H50	2.2960210911	6.0100654964	15.5081679977
H51	13.7148957738	6.0044445050	11.8563985090
O52	7.6503763940	7.2884397628	12.5672964561

O53	8.3617686211	7.2938177699	14.7787503803
O54	6.8339128257	8.4174451548	10.5377607536
O55	9.1593570653	8.4258758355	16.8134934729
H56	9.7965524724	6.1667935277	18.5588389697
H57	8.0122238662	-0.4116881346	13.6809891300
H58	9.3486516808	3.7751397416	19.2685234243

3

E (B3LYP-D3/LACVP**)(a.u.) = -2655.217634

ZPE (kcal mol^{-1}) = 272.307

G_{solv} (kcal mol^{-1})= -80.386

ΔH_{298} (kcal mol^{-1})= 21.491

ΔS_{298} (cal K^{-1} mol^{-1})= 203.924

Cartesian coordinates

Atom	x	y	z
Ru39	9.4802134197	5.7000519872	4.4122271276
S40	7.2388650303	5.7472203448	7.8100115418
S41	12.7351199892	5.5891165303	4.4189118039
N42	9.2390875918	7.2831615149	2.9680916092
N43	10.7739880353	4.2672663611	3.2721075782
O44	11.4662596925	6.4747793409	4.3767714224
N45	7.1912060753	5.7828500605	5.0004181448
C46	12.9041925985	3.1848531741	2.9835789666
H47	13.9685856393	3.1717340700	3.2052204415
O48	13.1076106994	5.1489096703	5.7713476386
O49	13.7965936466	6.2079665343	3.6107763856
C50	12.3043269224	2.2252110237	2.1630132935
H51	12.8935230460	1.4123332180	1.7461255413
N52	9.7377055903	4.1973726809	5.9041410819
N53	8.2785865541	4.6363704137	2.9402430670
C56	6.6275314965	6.2552854093	6.1344252137
C57	10.9499042849	2.3441306671	1.8493571204
H58	10.4757120150	1.6346849672	1.1772313942
C59	6.9990251659	5.0331719532	2.7671756556
C60	10.2077644706	3.3884044788	2.4101896100
C61	5.4268848825	6.9647442911	6.1172550245
H62	5.0143671658	7.3254462169	7.0526821380
O63	7.2278727542	4.2697509177	7.7426370214
C64	6.4644755648	5.8561602417	3.8557582617
C65	8.0770005167	3.1565142355	1.0610174952
H66	8.5225567470	2.4111910308	0.4088670405
C67	12.0900604693	4.1856494928	3.5004267387

C69	6.7760298755	3.6079352808	0.8423148243
H70	6.1907532700	3.2138596028	0.0155584593
C71	10.5090302043	4.4072636748	6.9907265641
H72	10.9795819248	5.3770521364	7.0871976147
C74	8.8115491008	3.6993947087	2.1176978926
C75	4.7667324181	7.1790507492	4.9054793505
H76	3.8567720069	7.7725981694	4.8687450163
C77	6.2232421631	4.5506397047	1.7079131203
H78	5.1944867090	4.8755932686	1.5868404784
C79	5.2657385923	6.5685115738	3.7611377075
H80	4.7504740663	6.6551101035	2.8091714254
C81	8.5132445800	8.3814082368	3.2661375059
H82	8.0587452047	8.4139687101	4.2492806571
C83	9.1226077864	3.0020772167	5.7468214168
H84	8.4806773199	2.8835148652	4.8805410964
O85	6.2620831884	6.3368583403	8.7569444747
O89	8.6076221156	6.3449342294	7.9845087607
C90	9.2874001452	1.9682246431	6.6565215296
H91	8.7802814168	1.0226853219	6.4870199864
C92	9.0154174629	9.3570417015	1.1291268701
H93	8.9273944628	10.1674350062	0.4094313110
C95	10.7074818984	3.4167390757	7.9470776095
H96	11.3375794715	3.6309772459	8.8051989694
C97	8.3753041877	9.4342693396	2.3674323736
H98	7.7771106678	10.2969912976	2.6468465635
C99	9.8651845635	7.2046481571	1.7740677211
H100	10.4503446341	6.3119808075	1.5776908424
C101	9.7755298124	8.2228120240	0.8312921069
H102	10.2964530975	8.1204889098	-0.1164921629
C103	10.0956264690	2.1760696856	7.7780841833
H104	10.2403576568	1.3835726877	8.5086831766
H59	9.1867477670	6.7765829934	6.5838510425
O61	9.4187491373	7.0915158961	5.6610670384

Reactant of H_2O coordination without buffer

E (B3LYP-D3/LACVP**)(a.u.) = -2655.656695

ZPE (kcal mol^{-1}) = 279.428

G_{solv} (kcal mol^{-1})= -161.563

ΔH_{298} (kcal mol^{-1})= 22.511

ΔS_{298} (cal K^{-1} mol^{-1})= 212.439

Cartesian coordinates

Atom	x	y	z

Ru39	9.3115540000000	5.5362750000000	4.5007250000000
S40	8.0432130000000	6.9685550000000	7.1322000000000
S41	12.4857560000000	5.3402180000000	4.9592530000000
N42	9.2648110000000	7.1908070000000	3.1492890000000
N43	10.7031180000000	4.3004620000000	3.3300100000000
O44	11.1868690000000	6.2234700000000	4.8963410000000
N45	7.1645620000000	5.7382810000000	4.9692700000000
C46	12.9153590000000	3.4211470000000	2.9698510000000
H47	13.9613720000000	3.4115250000000	3.2702570000000
O48	12.6272010000000	4.5768660000000	6.1994770000000
O49	13.6136380000000	6.1609450000000	4.5197590000000
C50	12.4392100000000	2.6540000000000	1.9030720000000
H51	13.1109530000000	2.0032670000000	1.3487350000000
N52	9.3550730000000	3.9133320000000	5.9061580000000
N53	8.1889030000000	4.5172710000000	2.9704200000000
C56	6.7290840000000	6.3417340000000	6.0842750000000
C57	11.0950920000000	2.7485440000000	1.5444040000000
H58	10.7110350000000	2.1740480000000	0.7062410000000
C59	6.8376680000000	4.6520220000000	2.9078800000000
C60	10.2435800000000	3.5852190000000	2.2740710000000
C61	5.3891890000000	6.5285470000000	6.3941270000000
H62	5.1051760000000	7.0285090000000	7.3184020000000
O63	8.2353990000000	6.0525630000000	8.2556640000000
C64	6.2481960000000	5.3106330000000	4.0644080000000
C65	8.1339450000000	3.2206160000000	0.9435980000000
H66	8.6669180000000	2.6407510000000	0.1956510000000
C67	12.0052240000000	4.2267620000000	3.6386940000000
C69	6.7602770000000	3.4216050000000	0.8385840000000
H70	6.2055290000000	3.0129500000000	-0.0020820000000
C71	9.9667820000000	4.0765840000000	7.0993480000000
H72	10.4262480000000	5.0362510000000	7.2904570000000
C74	8.8233470000000	3.7734640000000	2.0251750000000
C75	4.4418200000000	6.0784800000000	5.4697830000000
H76	3.3793310000000	6.2086310000000	5.6617540000000
C77	6.1016560000000	4.1260380000000	1.8434500000000
H78	5.0245590000000	4.2583520000000	1.8065270000000
C79	4.8758650000000	5.4733600000000	4.2906390000000
H80	4.1537890000000	5.1302030000000	3.5552650000000
C81	8.3581120000000	8.1784350000000	3.2997630000000
H82	7.6215390000000	8.0716490000000	4.0854320000000
C83	8.7561790000000	2.7293360000000	5.6250170000000
H84	8.2675140000000	2.6261060000000	4.6632270000000
O85	7.8028010000000	8.3820130000000	7.4199370000000
O89	9.1806270000000	6.8758730000000	6.0484550000000

C90	8.7570410000000	1.6765100000000	6.5289970000000
H91	8.2634280000000	0.7462980000000	6.2620770000000
C92	9.3428660000000	9.4375670000000	1.5102830000000
H93	9.3742060000000	10.3154490000000	0.8696920000000
C95	10.0039760000000	3.0606970000000	8.0492510000000
H96	10.5067270000000	3.2429130000000	8.9948520000000
C97	8.3604730000000	9.3091320000000	2.4926950000000
H98	7.6069500000000	10.0752440000000	2.6520100000000
C99	10.2228720000000	7.3155600000000	2.2069650000000
H100	10.9556170000000	6.5226400000000	2.1256280000000
C101	10.2897490000000	8.4216780000000	1.3694690000000
H102	11.0790890000000	8.4760500000000	0.6252090000000
C103	9.3923750000000	1.8413520000000	7.7646910000000
H104	9.4060010000000	1.0315230000000	8.4903440000000
H59	10.3147380000000	8.2645880000000	5.5567490000000
H60	10.4275450000000	9.7767740000000	5.4101600000000
O61	10.7136310000000	8.9445390000000	4.9996040000000

Transition state of H_2O coordination without buffer

E (B3LYP-D3/LACVP**)(a.u.) = -2655.622693

ZPE (kcal mol^{-1}) = 279.199

G_{solv} (kcal mol^{-1})= -165.081

ΔH_{298} (kcal mol^{-1})= 21.704

ΔS_{298} (cal K^{-1} mol^{-1})= 204.066

Cartesian coordinates

Atom	x	y	z
Ru39	9.3840233824	5.3772286180	4.2784051307
S40	7.9861996276	5.9471970952	7.6134896124
S41	12.5601894260	5.4994415208	4.5975090149
N42	9.3079604985	6.9190373371	2.7354675447
N43	10.7451421118	4.1114212969	3.2606257732
O44	11.1424197856	6.2370158461	4.6128509095
N45	7.3400635187	5.5528958792	4.8999858379
C46	12.9574564601	3.2193793075	3.0105827463
H47	14.0107619142	3.2634197183	3.2843429853
O48	12.9593068616	5.0651475075	5.9328877628
O49	13.4872576806	6.3072546610	3.8112508050
C50	12.4750015452	2.3144076358	2.0558304983
H51	13.1494684894	1.5976449507	1.5932395854
N52	9.4096247245	3.6845529369	5.6499962514
N53	8.2704775279	4.3747997004	2.8378860833
C56	6.9326164273	6.0281388418	6.0951374632

C57	11.1356174674	2.3709544987	1.6677311927
H58	10.7552136567	1.7068946837	0.8960024537
C59	6.9448587915	4.6463120002	2.7446925939
C60	10.2809019873	3.2911006221	2.2854989946
C61	5.6185674126	6.4652312481	6.2821165887
H62	5.3347256814	6.8622134720	7.2523239717
O63	7.7472889045	4.5743820127	8.1006549721
C64	6.4225751918	5.3885577612	3.8892375860
C65	8.1523564961	2.9161743455	0.9449607411
H66	8.6404871892	2.2236198909	0.2644078830
C67	12.0498578751	4.1020348001	3.5765265099
C69	6.8048074636	3.2458885446	0.7886384687
H70	6.2295457984	2.8142590878	-0.0266123166
C71	10.3262772223	3.5793899275	6.6369651474
H72	10.9781923754	4.4221819695	6.8007459325
C74	8.8695192525	3.5031211611	1.9888230294
C75	4.7044107897	6.3839991216	5.2350614978
H76	3.6830951891	6.7339268841	5.3650958206
C77	6.1871751074	4.1005559879	1.7042878892
H78	5.1233192646	4.3099546345	1.6308087231
C79	5.1050737650	5.8091179723	4.0266465727
H80	4.4007475018	5.6901408660	3.2083482867
C81	8.2975650865	7.8164486869	2.6791735177
H82	7.5575040050	7.7883978685	3.4671242539
C83	8.5247381855	2.6751959959	5.4658361480
H84	7.7751380679	2.7834899015	4.6917750779
O85	7.4703193971	7.0345752688	8.4708155952
O89	9.3884556272	6.1900235430	7.1410593075
C90	8.5472912773	1.5268187036	6.2461116724
H91	7.8141256607	0.7472562151	6.0599580578
C92	9.2337495124	8.8338702821	0.7133161178
H93	9.2063428985	9.5833208142	-0.0739949155
C95	10.4097373938	2.4508018826	7.4448866783
H96	11.1696800905	2.4159130364	8.2206895233
C97	8.2267349925	8.7792842573	1.6791599448
H98	7.3950992919	9.4782715273	1.6785511984
C99	10.2848747751	6.9685108637	1.8026429524
H100	11.0929212647	6.2507995613	1.8750808513
C101	10.2812824588	7.9131795998	0.7824378852
H102	11.0937984724	7.9182278575	0.0614356021
C103	9.5112254958	1.4028589317	7.2494866518
H104	9.5535218633	0.5107212129	7.8697012452
H59	9.5174188966	7.6691739892	6.1756828766
H60	10.0427577257	8.4250720314	4.9338291764

O61	9.2374316763	8.0208557810	5.3044573503

Product of H_2O coordination without buffer
E (B3LYP-D3/LACVP**)(a.u.) = -2655.647064
ZPE (kcal mol^{-1}) = 279.574
G_{solv} (kcal mol^{-1})= -166.762
ΔH_{298} (kcal mol^{-1})= 21.549
ΔS_{298} (cal K^{-1} mol^{-1})= 203.609

Cartesian coordinates

Atom	x	y	z
Ru39	9.4061024158	5.7154457437	4.3663982629
S40	7.6214701240	6.0164145746	7.8858000671
S41	12.6398175345	5.5955543172	4.6139551618
N42	9.3668838979	7.2147415443	2.8157054246
N43	10.7121297324	4.3377966130	3.3005575081
O44	11.2939616695	6.4133834090	4.7128762925
N45	7.2675203258	5.8361003249	5.0388685530
C46	12.8681034986	3.3158246591	3.0167096706
H47	13.9246930473	3.3000628055	3.2791476595
O48	13.0772075092	5.0589114219	5.9013043364
O49	13.5956728537	6.3766129604	3.8290289308
C50	12.3193766170	2.4149447377	2.0968519612
H51	12.9433257351	1.6497428447	1.6409947088
N52	9.5556993530	4.1567431802	5.8407088922
N53	8.2285442214	4.6772117273	2.9492387341
C56	6.7754344119	6.2485657117	6.2340103787
C57	10.9744642737	2.5319059212	1.7424141530
H58	10.5390810546	1.8679270015	1.0005443289
C59	6.9189511656	5.0021011122	2.8408423313
C60	10.1899066121	3.5136072262	2.3579971257
C61	5.4577725744	6.6993767546	6.3563283399
H62	5.1056944110	7.0213924026	7.3300171286
O63	7.5936340974	4.5549919896	8.0724225007
C64	6.3957344698	5.7392345483	3.9850441557
C65	8.0556514951	3.2559603491	1.0246637522
H66	8.5208063324	2.5548868783	0.3369099789
C67	12.0211183746	4.2613968932	3.5785327165
C69	6.7253903047	3.6437198770	0.8597488280
H70	6.1393261832	3.2504152568	0.0328082190
C71	10.4072810114	4.2381880745	6.8849929478
H72	10.9294210555	5.1721523100	7.0313072532
C74	8.7900113785	3.7950139391	2.0807573409

C75	4.6137724559	6.7236527940	5.2492662155
H76	3.5996748295	7.1065318128	5.3324650255
C77	6.1438107733	4.5112586698	1.7826572211
H78	5.0933710789	4.7738360235	1.7004103049
C79	5.0808193971	6.1971161413	4.0513107640
H80	4.4343495121	6.1379048828	3.1812053280
C81	8.4226297406	8.1839423389	2.8275464419
H82	7.7123224892	8.1792846998	3.6459210503
C83	8.8396596576	3.0201458918	5.6554189606
H84	8.1349121405	2.9931437897	4.8318440767
O85	6.8049034970	6.7978098010	8.8351173816
O89	9.0149757023	6.5848231994	7.7521030290
C90	8.9830747946	1.9217484303	6.4901017824
H91	8.3924214520	1.0299160653	6.3005199213
C92	9.3489371842	9.1805432198	0.8479001040
H93	9.3445168489	9.9511884923	0.0809384865
C95	10.5984192459	3.1706416191	7.7564861429
H96	11.3000290882	3.2824669203	8.5781563121
C97	8.3820867806	9.1768406452	1.8558545374
H98	7.6049145036	9.9344194247	1.9063209380
C99	10.3058354865	7.2141429745	1.8431471317
H100	11.0575765722	6.4344494107	1.8663603730
C101	10.3265177082	8.1825788761	0.8463678856
H102	11.1045332328	8.1471786687	0.0889829001
C103	9.8854877149	1.9905601464	7.5558754253
H104	10.0213987409	1.1399558781	8.2199504998
H59	9.2189114383	7.2456064048	6.4997750558
H60	9.9951104600	8.0824711291	5.3343557198
O61	9.2188897522	7.5014293927	5.4913950725

Reactant of H_2O coordination with buffer

E (B3LYP-D3/LACVP**)(a.u.) = -3299.412671

ZPE (kcal mol^{-1}) = 305.198

G_{solv} (kcal mol^{-1})= -85.245

ΔH_{298} (kcal mol^{-1})= 25.042

ΔS_{298} (cal K^{-1} mol^{-1})= 221.030

Cartesian coordinates

Atom	x	y	z
Ru39	10.0666785985	5.7299809892	4.7915295196
S40	9.2026598491	7.9499093276	8.0331212220
S41	13.2617553764	5.2325097733	4.8727976446
N42	10.0719458970	6.8597242069	2.8714384684

N43	11.1267318928	4.4233818961	3.5199155632
O44	12.1335762309	6.2120528891	5.2666694418
N45	8.2240171538	6.0074587018	6.1569994558
C46	13.1568127319	3.6668017486	2.5162527062
H47	14.2437810281	3.6418895865	2.5254727187
O48	13.5377680242	4.2195202879	5.9009886763
O49	14.4122793057	5.9709584163	4.3326590989
C50	12.4080714217	3.0243615291	1.5244779298
H51	12.9129164659	2.4831401034	0.7279832933
N52	10.3451010909	4.2494388670	6.3402937843
N53	8.5926018058	4.5898235836	3.9285709205
C56	8.1068390763	6.5992848902	7.3673778377
C57	11.0091451840	3.0724005188	1.5566754006
H58	10.4254834548	2.5658216385	0.7938581111
C59	7.3310091107	4.6209978722	4.3971698001
C60	10.3788499115	3.7821533249	2.5799486140
C61	6.9891414650	6.3959337685	8.1842119628
H62	6.9456809204	6.8821139280	9.1523732201
O63	8.9786945970	7.9249808322	9.4924104066
C64	7.1742528217	5.2582025791	5.7104052656
C65	7.9372699931	3.2834508925	2.0391003541
H66	8.1882860589	2.7359101830	1.1359051549
C67	12.4607617956	4.3654060167	3.4955411889
C69	6.6028834343	3.3987759608	2.4558804188
H70	5.8086980693	2.9648432325	1.8542659382
C71	10.9773503597	4.6066597520	7.4803735905
H72	11.2873791956	5.6385383440	7.5635922816
C74	8.9285714734	3.8838872699	2.8067910187
C75	5.9402823694	5.5973813079	7.7417944250
H76	5.0704310024	5.4289506391	8.3714810668
C77	6.2948536954	4.0389178329	3.6518256436
H78	5.2663931708	4.1030187494	3.9907750204
C79	6.0248921872	5.0373568982	6.4742178399
H80	5.2218939358	4.4156727298	6.0942245911
C81	9.0078118258	7.0193948781	2.0618070724
H82	8.0838230296	6.5371914160	2.3505243000
C83	9.9563338025	2.9626421244	6.1729727444
H84	9.4659019808	2.6923930083	5.2460090066
O85	8.6602651355	9.1567662553	7.3366951487
O89	10.6291809508	7.6656852042	7.6720812153
C90	10.1755779006	2.0004268564	7.1506657935
H91	9.8470216415	0.9806684797	6.9714728068
C92	10.2499810264	8.5075327493	0.6359574504
H93	10.3170299345	9.1580599876	-0.2327779017

C95	11.2167090800	3.6918651303	8.5003530752
H96	11.7231561860	4.0295033755	9.4004581883
C97	9.0649216542	7.8353670051	0.9335707136
H98	8.1772144685	7.9488654381	0.3179201231
C99	11.2184224167	7.5230656626	2.5986009040
H100	12.0280541416	7.4121413892	3.3068764727
C101	11.3466483318	8.3440803241	1.4872566960
H102	12.2907481743	8.8530704723	1.3106128668
C103	10.8127507192	2.3669512149	8.3391601642
H104	10.9935309835	1.6314843092	9.1195357576
H59	10.5159397078	7.7672843758	6.0472388406
H60	7.1401917043	9.2393650310	6.3668539015
O61	10.0771852753	7.6670207312	5.1633219005
P69	6.7260166329	8.7089079903	4.2777710357
O70	5.8930044292	9.7904662166	3.4252008657
O71	6.4169762458	7.3047536977	3.8711507394
O72	6.3520025893	9.0563640858	5.7994124169
O73	8.2317380280	9.1992984537	4.0953081439
H74	8.9188873479	8.6305199942	4.5592509545
H75	5.3719797126	9.3680309970	2.7151790843

Transition state of H_2O coordination with buffer

E (B3LYP-D3/LACVP**)(a.u.) = -3299.363606

ZPE (kcal mol^{-1}) = 303.536

G_{solv} (kcal mol^{-1})= -94.190

ΔH_{298} (kcal mol^{-1})= 24.922

ΔS_{298} (cal K^{-1} mol^{-1})= 219.907

Cartesian coordinates

Atom	x	y	z
Ru39	10.1502757614	5.2135813442	4.7671643560
S40	9.4491810485	7.0667899051	7.7995450159
S41	13.2842943160	4.8717307705	4.7967989065
N42	10.0578128066	6.3456435058	2.8766008448
N43	11.1985975890	3.7922442549	3.5842212890
O44	12.0387850853	5.8651243112	4.8325406866
N45	8.2813582087	5.4622369015	5.8698760984
C46	13.2793249826	2.8759842626	2.8174679597
H47	14.3639355649	2.8453373256	2.9035743739
O48	13.5557064823	4.2277233809	6.0779914707
O49	14.3730400511	5.5577582814	4.1038205406
C50	12.5885253290	2.1434764340	1.8430603588
H51	13.1317242841	1.4958904628	1.1589833933

N52	10.4130111449	3.7560274279	6.3899588690
N53	8.7016369719	4.1151841546	3.7150065567
C56	8.1754520220	5.9680420319	7.1220509787
C57	11.2045236450	2.2781183464	1.7317077184
H58	10.6576917837	1.7407218543	0.9614080137
C59	7.3940240559	4.2655006437	4.0228935962
C60	10.5231532762	3.1170222573	2.6203101651
C61	7.0136915147	5.8509994934	7.8771055714
H62	6.9945176556	6.2667892451	8.8816504056
O63	9.5758314907	6.7231725309	9.2286579420
C64	7.1726951112	4.8897322929	5.3188722367
C65	8.1364948400	2.8000687099	1.7984232506
H66	8.4485558744	2.1974241134	0.9501333028
C67	12.5357114040	3.6950943512	3.6518496431
C69	6.7820822525	3.0549215155	2.0360665890
H70	6.0269177105	2.6726666320	1.3539403522
C71	11.1448129946	3.9811095293	7.5032793093
H72	11.6034983879	4.9525720363	7.5955307179
C74	9.0829513855	3.3405621307	2.6672339419
C75	5.8926328974	5.2354586362	7.3238444619
H76	4.9767021864	5.1291507328	7.8996304889
C77	6.4021064756	3.7604383660	3.1755603292
H78	5.3524669917	3.9154400413	3.4089904015
C79	5.9677580202	4.7774596813	6.0148993983
H80	5.1123172559	4.3031418092	5.5453331382
C81	8.8783140518	6.8397670903	2.4520100840
H82	7.9961575798	6.6685236070	3.0593159610
C83	9.8288793651	2.5414180881	6.2276808838
H84	9.2454276311	2.3708022172	5.3312959480
O85	8.9000977507	8.4205233570	7.5144976204
O89	10.6668611082	6.7558266044	6.9773406982
C90	9.9550839260	1.5294433780	7.1690321349
H91	9.4665134200	0.5760089630	6.9889371065
C92	9.9319351644	7.8876715176	0.5608911785
H93	9.8847734538	8.4985889913	-0.3373663987
C95	11.3064013650	3.0100566244	8.4877948629
H96	11.9057897413	3.2469776751	9.3625744394
C97	8.7821731628	7.6120878342	1.2988164620
H98	7.8095695953	8.0000416628	1.0114769248
C99	11.1780317108	6.6136035074	2.1723173374
H100	12.1172395738	6.2429859326	2.5547546493
C101	11.1510316004	7.3746965083	1.0103849423
H102	12.0817368871	7.5640885655	0.4822373551
C103	10.7065368604	1.7632608713	8.3248879825

H104	10.8215376456	0.9869401146	9.0776821744
H59	10.4188719710	8.1635055155	5.6649594013
H60	7.0654243282	8.7207778793	6.5559282096
O61	10.1456241667	8.0784603654	4.7393628284
P69	6.4188220667	8.4814968316	4.4888133108
O70	5.1781929497	9.4694680665	4.0521605281
O71	6.1361192576	7.1076817010	3.9128360612
O72	6.2263610498	8.4819183325	6.1176416955
O73	7.7444377029	9.1690675455	4.1901860283
H74	9.2448323120	8.4910088964	4.6528215868
H75	4.6627552005	9.0524781187	3.3385736809

Product of H_2O coordination with buffer

E (B3LYP-D3/LACVP**)(a.u.) = -3299.412671

ZPE (kcal mol^{-1}) = 305.198

G_{solv} (kcal mol^{-1})= -85.245

ΔH_{298} (kcal mol^{-1})= 25.042

ΔS_{298} (cal K^{-1} mol^{-1})= 221.030

Cartesian coordinates

Atom	x	y	z
Ru39	10.0666785985	5.7299809892	4.7915295196
S40	9.2026598491	7.9499093276	8.0331212220
S41	13.2617553764	5.2325097733	4.8727976446
N42	10.0719458970	6.8597242069	2.8714384684
N43	11.1267318928	4.4233818961	3.5199155632
O44	12.1335762309	6.2120528891	5.2666694418
N45	8.2240171538	6.0074587018	6.1569994558
C46	13.1568127319	3.6668017486	2.5162527062
H47	14.2437810281	3.6418895865	2.5254727187
O48	13.5377680242	4.2195202879	5.9009886763
O49	14.4122793057	5.9709584163	4.3326590989
C50	12.4080714217	3.0243615291	1.5244779298
H51	12.9129164659	2.4831401034	0.7279832933
N52	10.3451010909	4.2494388670	6.3402937843
N53	8.5926018058	4.5898235836	3.9285709205
C56	8.1068390763	6.5992848902	7.3673778377
C57	11.0091451840	3.0724005188	1.5566754006
H58	10.4254834548	2.5658216385	0.7938581111
C59	7.3310091107	4.6209978722	4.3971698001
C60	10.3788499115	3.7821533249	2.5799486140
C61	6.9891414650	6.3959337685	8.1842119628
H62	6.9456809204	6.8821139280	9.1523732201

O63	8.9786945970	7.9249808322	9.4924104066
C64	7.1742528217	5.2582025791	5.7104052656
C65	7.9372699931	3.2834508925	2.0391003541
H66	8.1882860589	2.7359101830	1.1359051549
C67	12.4607617956	4.3654060167	3.4955411889
C69	6.6028834343	3.3987759608	2.4558804188
H70	5.8086980693	2.9648432325	1.8542659382
C71	10.9773503597	4.6066597520	7.4803735905
H72	11.2873791956	5.6385383440	7.5635922816
C74	8.9285714734	3.8838872699	2.8067910187
C75	5.9402823694	5.5973813079	7.7417944250
H76	5.0704310024	5.4289506391	8.3714810668
C77	6.2948536954	4.0389178329	3.6518256436
H78	5.2663931708	4.1030187494	3.9907750204
C79	6.0248921872	5.0373568982	6.4742178399
H80	5.2218939358	4.4156727298	6.0942245911
C81	9.0078118258	7.0193948781	2.0618070724
H82	8.0838230296	6.5371914160	2.3505243000
C83	9.9563338025	2.9626421244	6.1729727444
H84	9.4659019808	2.6923930083	5.2460090066
O85	8.6602651355	9.1567662553	7.3366951487
O89	10.6291809508	7.6656852042	7.6720812153
C90	10.1755779006	2.0004268564	7.1506657935
H91	9.8470216415	0.9806684797	6.9714728068
C92	10.2499810264	8.5075327493	0.6359574504
H93	10.3170299345	9.1580599876	-0.2327779017
C95	11.2167090800	3.6918651303	8.5003530752
H96	11.7231561860	4.0295033755	9.4004581883
C97	9.0649216542	7.8353670051	0.9335707136
H98	8.1772144685	7.9488654381	0.3179201231
C99	11.2184224167	7.5230656626	2.5986009040
H100	12.0280541416	7.4121413892	3.3068764727
C101	11.3466483318	8.3440803241	1.4872566960
H102	12.2907481743	8.8530704723	1.3106128668
C103	10.8127507192	2.3669512149	8.3391601642
H104	10.9935309835	1.6314843092	9.1195357576
H59	10.5159397078	7.7672843758	6.0472388406
H60	7.1401917043	9.2393650310	6.3668539015
O61	10.0771852753	7.6670207312	5.1633219005
P69	6.7260166329	8.7089079903	4.2777710357
O70	5.8930044292	9.7904662166	3.4252008657
O71	6.4169762458	7.3047536977	3.8711507394
O72	6.3520025893	9.0563640858	5.7994124169
O73	8.2317380280	9.1992984537	4.0953081439

H74	8.9188873479	8.6305199942	4.5592509545
H75	5.3719797126	9.3680309970	2.7151790843
